# 3D-printed epifluidic electronic skin for machine learning–powered multimodal health surveillance

**DOI:** 10.1126/sciadv.adi6492

**Published:** 2023-09-13

**Authors:** Yu Song, Roland Yingjie Tay, Jiahong Li, Changhao Xu, Jihong Min, Ehsan Shirzaei Sani, Gwangmook Kim, Wenzheng Heng, Inho Kim, Wei Gao

**Affiliations:** Andrew and Peggy Cherng Department of Medical Engineering, California Institute of Technology, Pasadena, CA 91125, USA.

## Abstract

The amalgamation of wearable technologies with physiochemical sensing capabilities promises to create powerful interpretive and predictive platforms for real-time health surveillance. However, the construction of such multimodal devices is difficult to be implemented wholly by traditional manufacturing techniques for at-home personalized applications. Here, we present a universal semisolid extrusion–based three-dimensional printing technology to fabricate an epifluidic elastic electronic skin (e^3^-skin) with high-performance multimodal physiochemical sensing capabilities. We demonstrate that the e^3^-skin can serve as a sustainable surveillance platform to capture the real-time physiological state of individuals during regular daily activities. We also show that by coupling the information collected from the e^3^-skin with machine learning, we were able to predict an individual’s degree of behavior impairments (i.e., reaction time and inhibitory control) after alcohol consumption. The e^3^-skin paves the path for future autonomous manufacturing of customizable wearable systems that will enable widespread utility for regular health monitoring and clinical applications.

## Teaser

A 3D-printed microfluidic wearable sensor could perform multimodal health monitoring and predict behavior impairments.

## INTRODUCTION

Maintaining a well-balanced lifestyle and effective recognition of premedical symptoms to obtain early intervention is paramount to sustain one’s physical well-being and attain longevity. With the advent of wearable technology, traditional healthcare practices are rapidly changing their course through the implementation of personalized medicine and digital health (*1*–*3*). Skin-interfaced wearable devices that deliver intimate details relating to the users’ health status in real-time are deemed integral enablers to this endeavor (*4*–*7*). Real-time tracking of vital signs such as heart rate and body temperature from the skin provides insightful information on the physiological condition of the human body. On the other side, in situ microfluidic sampling and analysis of sweat, a key noninvasively accessible body fluid, could offer rich biomolecular information closely associated with our health state (*8*–*15*). To this end, there is an unprecedented need to develop multimodal wearable systems with both molecular sensing and vital sign tracking capabilities for more comprehensive information of our bodily responses (*14*, *16*). Such multimodal data, when coupled with modern data analysis approaches (such as machine learning), will enable numerous practical health surveillance and clinical applications (*17*, *18*).

Despite the high demand, fabrication and integration of transdisciplinary modules for such wearable device involve processes that use highly customizable materials and designs. For example, patterned nanomaterials and composites are often used to increase the active surface area of electrochemical sweat sensors for enhanced sensing capabilities (*13*, *14*, *19*); use of various biorecognition molecules (e.g., enzymes and ionophores) in a polymer matrix is often necessary for selective detection of specific biomarkers (e.g., metabolites and ions) (*8*, *9*). Polymeric hydrogels are commonly patterned on the electrodes for transdermal delivery of the nicotinic agents (e.g., pilocarpine and carbachol) via iontophoresis for autonomous sweat induction, while microfluidic channels that regulate and sample the sweat flow are typically fabricated through polymer molding or laser cutting of plastic films (*10*, *15*, *20*). Conversely, three-dimensional (3D) micro/nanostructures are often required for highly sensitive pressure and strain sensing with compressible and stretchable functionalities (*21*, *22*). Hence, the incorporation of such complex fabrication, which encompasses a diverse range of materials and processes, traditionally requires the combination of a series of conventional cleanroom facilities and manufacturing technologies. Moreover, complementary laborious interventions such as manual deposition and assembly are usually performed at a laboratory scale. The development of a scalable and customizable prototyping and fabrication method that caters to the aforementioned fabrication needs would be vital for the future widespread implementation of multimodal wearable sensors in personalized healthcare but has not been realized yet.

**Fig. 1.**
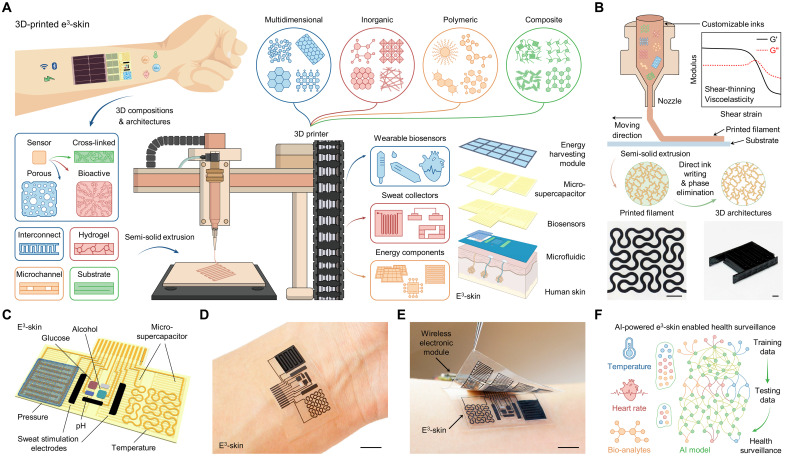
SSE-based 3D-printed e^3^-skin. (**A**) Schematic illustration of the SSE-based 3D printing that features highly customizable inks based on versatile materials to construct all main building blocks of the wearable e^3^-skin with multimodal sensing and power management capabilities. (**B**) Schematic illustration of SSE printing procedures to prepare 2D and 3D architectures. Top right inset, typical rheological properties of printable inks; bottom, optical images of as-printed 2D and 3D MXene architectures. G′, storage modulus; G″, loss modulus. Scale bars, 2 mm. (**C**) Schematic illustration of the 3D-printed e^3^-skin that comprises multiplexed biophysical and biochemical sensors for pulse waveform, temperature, and sweat biomarker monitoring, a microfluidic iontophoretic module for localized automatic sweat induction and sampling, and MSCs for energy storage. (**D** and **E**) Optical images of an e^3^-skin (D) and a fully assembled wireless e^3^-skin system (E) worn on a human subject. Scale bars, 1 cm. (**F**) Machine learning–powered multimodal e^3^-skin for personalized health surveillance. AI, artificial intelligence.

To address these challenges, here, we present an epifluidic elastic electronic skin (e^3^-skin) with multimodal physiochemical sensing capabilities, which is constructed exclusively using a highly adaptable and versatile semisolid extrusion (SSE)–based 3D-printing technology involving direct ink writing and selective phase elimination (Fig. 1A). This 3D-printed e^3^-skin, coupled with machine learning, enables remote multimodal personalized health assessment. To prepare the e^3^-skin with optimal performance for on-body biosensing, epifluidic modulation, and energy efficiency, functional inks comprising various multidimensional nanomaterials, polymers, and hydrogels are custom-tailored to pattern all multidimensional architectures in the wearable system with high precision (figs. S1 and S2). All inks were formulated to fulfill the desirable rheological properties for SSE, which necessitate suitable viscoelasticity and shear-thinning behaviors as instructed by the choice of materials combination (Fig. 1B, fig. S1, and table S1) (*23*, *24*). A phase elimination strategy, which involves the selective removal of the sacrificial component in the ink (*25*–*28*), was used to transform as-printed 3D filaments into porous architectures to enhance the performance. This technology enables low-cost customizable prototyping of sustainable and wearable multifunctional physiochemical sensing systems via a simple process, ideally suitable for remote healthcare surveillance (fig. S2).

The SSE-based 3D-printed e^3^-skin is composed of an array of electrochemical sweat biosensors (e.g., glucose, alcohol, and pH sensors) and biophysical sensors (e.g., temperature and pulse sensors), a pair of hydrogel-coated iontophoresis electrodes for localized sweat induction, a microfluidics for efficient sweat sampling, and a micro-supercapacitor (MSC) as energy storage module interfaced with an energy-harvesting device (e.g., solar cell) for sustainable wearable operation (Fig. 1C). Further integrated with wireless electronic module, the e^3^-skin could perform prolonged physiochemical data collection from the daily activities (Fig. 1, D and E). Such multimodal data collection, coupled with machine learning–based data analytics, opens the door to a wide range of personalized healthcare applications in the era of digital health. As an exemplar, we demonstrate that simultaneous monitoring of the pulse waveform, temperature, and alcohol levels using a machine learning–coupled e^3^-skin is able to accurately predict an individual’s behavior response (Fig. 1F).

## RESULTS

### 3D-printed biophysical sensors

**Fig. 2.**
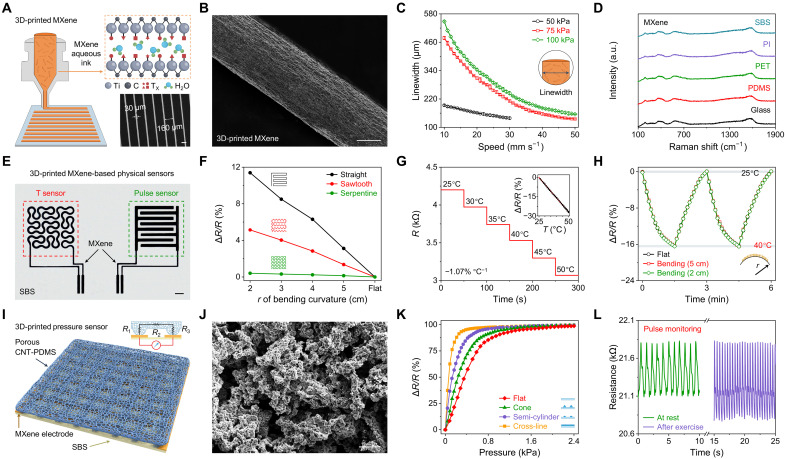
Design and characterization of 3D-printed interconnects and biophysical sensors. (**A**) Schematic illustration of high-precision SSE-based 3D printing using an aqueous MXene ink. Inset, microscopic image displaying an array of 3D-printed MXene filaments with narrow gaps down to 30 μm. Scale bar, 100 μm. (**B**) Scanning electron microscopy (SEM) image of a 3D-printed MXene filament. Scale bar, 100 μm. (**C**) Dependence of the linewidth of the 3D-printed MXene on printing speed and pressure. Error bars represent the SD from five measurements. (**D**) Raman spectra of 3D-printed MXene on various substrates. SBS, styrene-butadiene-styrene; PDMS, polydimethylsiloxane; PET, polyethylene terephthalate; PI, polyimide. (**E**) Optical image of MXene-based temperature and pulse sensors. Scale bar, 2 mm. (**F**) Resistive change of temperature sensors based on different designs under varying bending curvatures. (**G**) Dynamic response of an MXene-based temperature sensor under varying temperatures (*T*). Inset, calibration plot within the physiological temperature range. Error bars represent the SD from five measurements. (**H**) Responses of the temperature sensor under mechanical deformations and periodically changing temperature. (**I**) Schematic illustration of the 3D-printed pressure sensor consisting of an interdigital MXene electrode and a porous CNT-PDMS active layer. (**J**) SEM image of the porous 3D-printed CNT-PDMS. Scale bar, 50 μm. (**K**) Resistive responses of the pressure sensors based on different surface architectures under applied pressure. (**L**) Real-time monitoring of the radial pulse of a human subject using the 3D-printed pulse sensor at rest and after exercise. a.u., arbitrary units.

The interconnects and biophysical sensors in the e^3^-skin were prepared primarily based on high-precision SSE with an aqueous Ti_3_C_2_T_x_ (MXene) ink (Fig. 2A, figs. S3 and S4, and movie S1). Owing to the bidimensionality with high aspect ratio, negatively charged surfaces, and intrinsic hydrophilicity, mono- to few-layer MXene nanosheets, with an average lateral size of 2.63 μm (fig. S5), feature strong electrostatic repulsion properties, making them highly dispersible and stable in water (*29*–*31*). The printed linewidth of the MXene filaments can be modulated by tuning the pressure and speed of the extrusion printer. Uniform arrays of intricate lines could reach a minimum average linewidth of 160 μm and a line gap down to 10 μm (Fig. 2, B and C, and fig. S4). The MXene filaments can be readily printed onto a variety of flexible substrates, as evidenced by the identical MXene fingerprints in Raman spectra (Fig. 2D).

In addition to its applications as interconnects, MXene was also used as an active material for wearable temperature sensing (Fig. 2E). In the e^3^-skin, an MXene-based temperature sensor was patterned by adopting a strain-insensitive serpentine design to withstand the stress during daily wear (Fig. 2F). With a linewidth of 350 μm, it exhibits a negative temperature coefficient behavior with a sensitivity of −1.07% °C^−1^ across a physiologically relevant range of 25° to 50°C (Fig. 2G). Other printed temperature sensors with different linewidths showed similar sensitivities (fig. S6). Stable temperature sensing performance with fast response time was observed during mechanical bending tests and upon placing onto human skin (Fig. 2H and fig. S7).

The e^3^-skin’s pulse monitoring capability was based on a pressure sensor composed of an interdigital MXene electrode and a porous carbon nanotube (CNT)–polydimethylsiloxane (PDMS) foam as the active sensing component (Fig. 2I). The latter was prepared via 3D printing with a customizable ink containing homogeneously mixed PDMS, CNT, and finely grounded salt microparticles, followed by selective salt removal to form the porous structure with an average pore size of 30 μm (Fig. 2J and fig. S8A). Such high porosity plays a crucial role to realize high-pressure sensitivity (fig. S8, B and C) and can be optimized by controlling the size of salt microparticles and varying the compositional ratio of CNT-PDMS and salt to fulfill the rheological criteria for SSE with robust mechanical stability (figs. S9 to S11). Although multiple 3D-printed surface architectures (e.g., cone, semi-cylinder, and cross-line architectures) were able to improve the sensitivity (Fig. 2K and fig. S12), pressure sensor based on a cross-line architecture yielded the highest sensitivity due to the increased contact area and enabled reliable radial pulse monitoring on human subjects (Fig. 2L). The printed CNT-PDMS foam is mechanically resilient and superelastic, demonstrating repetitive and reproducible resistance changes under 20,000 pressing-releasing cycles (fig. S13).

### 3D-printed biochemical sensors

**Fig. 3.**
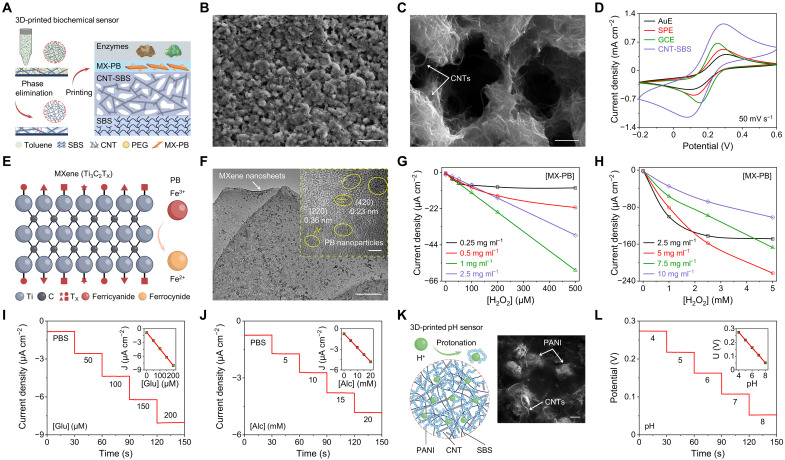
Design and characterizations of the 3D-printed biochemical sensors. (**A**) Schematic illustration of the design and SSE-based 3D-printing process of the enzymatic sensors. PEG, polyethylene glycol; MX-PB, MXene–Prussian blue. (**B**) SEM image of the porous CNT-SBS after PEG dissolution. Scale bar, 10 μm. (**C**) Magnified SEM image of the porous CNT-SBS with exposed CNTs. Scale bar, 1 μm. (**D**) Cyclic voltammetry (CV) scans of a gold electrode (AuE), an SPE, a GCE, and a 3D-printed CNT-SBS electrode in a solution containing 5 mM [Fe(CN)_6_]^3−^ and 0.1 M KCl. (**E**) Schematic illustration of in situ reduction of PB onto MXene. (**F**) TEM image of an MX-PB film. Scale bar, 500 nm. Inset, magnified TEM image revealing the PB nanoparticles as circled in yellow. Scale bar, 5 nm. (**G** and **H**) Amperometric calibration plots of CNT-SBS electrodes with varying MX-PB loadings in hydrogen peroxide (H_2_O_2_) solutions with low (0 to 500 μM) (G) and high (0 to 5 mM) (H) concentrations. (**I** and **J**) Amperometric responses of the glucose sensor (I) and alcohol sensor (J). Insets, the corresponding calibration plots. PBS, phosphate-buffered saline; Glu, glucose; Alc, alcohol. Error bars represent the SD from 10 sensors. (**K**) Schematic illustration of the working mechanism of the 3D-printed CNT-SBS-PANI–based pH sensor and the corresponding SEM image. Scale bar, 1 μm. (**L**) Open-circuit potential response of a pH sensor in Mcllvaine’s buffer. Inset, the corresponding calibration plot. Error bars represent the SD from 10 sensors.

The proposed SSE-based 3D-printing technology was successfully implemented to prepare a variety of electrochemical biosensors on the e^3^-skin (Fig. 3A). For example, enzymatic biosensors were fabricated through sequential printing of porous CNT–styrene-butadiene-styrene (CNT-SBS) as the working electrode (WE), MXene–Prussian blue (MX-PB) as the redox mediator, and the bioactive polymer [e.g., chitosan and bovine serum albumin (BSA)] loaded with enzymes [e.g., glucose oxidase (GOx) and alcohol oxidase (AOx)] as the target recognition element. Considering that high electrochemical activity and active surface area are highly desired for electrochemical WEs, CNT-SBS was chosen over multiple other carbon-based composite inks [e.g., graphite-SBS and carbon black (CB)–SBS] that fits the rheological criteria for SSE (fig. S14). Uniformly distributed porous structures were introduced via phase elimination of polyethylene glycol (PEG) within a printed CNT-SBS-PEG composite (with optimized CNT and PEG ratio) to maximize the active surface area of the WE (Fig. 3, B and C, and fig. S15). The optimized 3D-printed porous CNT-SBS WE displayed a superior electrochemical performance compared to other commercial WEs such as glassy carbon electrode (GCE), screen-printed carbon electrode (SPE), and Au electrode (AuE) (Fig. 3D), and was able to detect ultralow-level uric acid (UA) in sweat through direct oxidization (fig. S16).

To prepare the enzymatic electrochemical sensors, PB was chosen as the redox mediator as it enables low-voltage operation (~0 V versus reference electrode) and minimizes the interferences from other electroactive molecules (*32*). Here, we introduced an in situ reduction strategy to formulate high-performance printable MX-PB as a mediator layer on top of the CNT-SBS WE (Fig. 3E, note S1, and fig. S17). Transmission electron microscopy (TEM) images depict uniformly deposited PB nanoparticles over the MXene film surface with an average size of ~8 nm (Fig. 3F and fig. S18). The concentration of MX-PB was tailored with desired sensitivity and linear operating range according to the levels of target biomarkers in sweat (Fig. 3, G and H). In particular, because of the charge transfer properties including electron hopping and counter-ion movement within the PB layer, MX-PB (1 mg ml^−1^) resulted in a high sensitivity, while increased concentration (10 mg ml^−1^) of MX-PB led to a decreased sensitivity and wide linear operating window due to the slower charge transfer kinetics (*33*). The incorporation of MX-PB in the mediator layer substantially enhanced both sensitivity and detection limit as compared to neat PB due to the synergistic hybridized network that enhances electronic coupling for interfacial electron transfer (fig. S19).

Cocktails comprising enzymes (i.e., GOx and AOx) and bioactive polymers (i.e., chitosan and BSA) were then directly printed on the MX-PB mediator layer to prepare glucose and alcohol sensors suitable for wearable sweat analysis (fig. S20). Figure 3 (I and J) shows the representative amperometric responses of the optimized glucose and alcohol sensors, measured at physiologically relevant concentrations between 0 to 200 μM and 0 to 20 mM, respectively. A linear relationship between the current and analyte’s concentration with sensitivities of 0.036 μA cm^−2^ μM^−1^ for glucose sensor and 0.204 μA cm^−2^ mM^−1^ for alcohol sensor was observed. Considering that sweat alcohol concentration after alcohol intake can be as high as tens of mM, a polyurethane (PU) diffusion–limiting membrane was introduced to improve the current stability and widen the linear range (fig. S21), and the developed alcohol sensor demonstrated high stability over prolonged periods and multiple cycles of measurements (fig. S22). Both enzymatic sensors showed high reproducibility (fig. S23) and selectivity over other metabolites typically found in sweat (fig. S24) and were able to reliably detect the analyte-level changes when sampled at physiological sweat rates (fig. S25).

The pH sensor on the e^3^-skin was designed on the basis of a CNT-SBS-polyaniline (PANI) electrode printed with an ink made of PANI powder and CNT-SBS (fig. S26A). pH was measured as a function of potential changes caused by protonation/deprotonation on the PANI surface (Fig. 3K). A near-Nernstian sensitivity of 55.6 mV pH^−1^ with high reproducibility over a physiologically relevant pH range of 4 to 8 was obtained (Fig. 3L and fig. S26, B and C). To accurately quantify sweat glucose and alcohol levels, real-time sensor calibrations were performed on the basis of the simultaneously obtained pH and temperature information to compensate the influence of pH and temperature on the enzymatic reactions (figs. S27 and S28).

### 3D-printed microfluidics for biofluid extraction, sampling, and multiplexed analysis

**Fig. 4.**
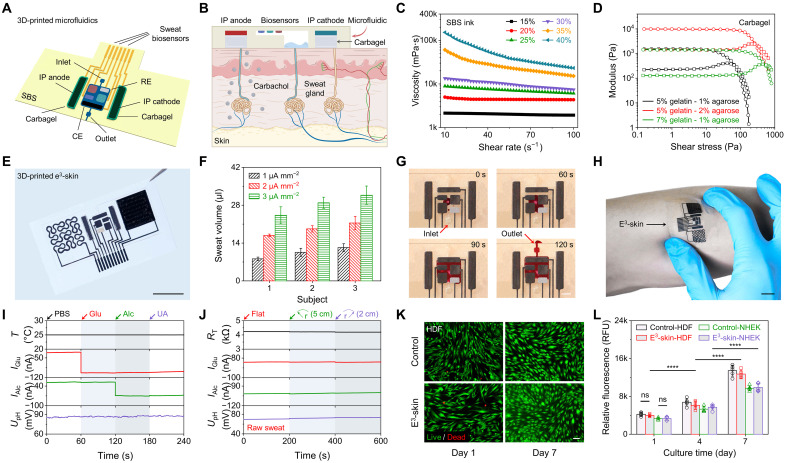
Design and characterization of the 3D-printed microfluidics for sweat induction, sampling, and multiplexed analysis. (**A**) Schematic illustration of the SSE-based 3D-printed microfluidics. IP, iontophoresis; RE, reference electrode; CE, counter electrode. (**B**) Schematic of the microfluidics-based localized iontophoretic sweat induction through transdermal delivery of muscarinic agent carbachol. (**C**) Viscosity plotted as a function of shear rate for SBS inks with varying concentrations. (**D**) Storage (circles) and loss (squares) modulus of gelatin-agarose hydrogels containing carbachol (carbagel) plotted as a function of shear stress. (**E**) Optical image of a 3D-printed e^3^-skin with an integrated microfluidics module. Scale bar, 1 cm. (**F**) Plots of sampling sweat volumes from three subjects after administering different carbachol dosages. (**G**) Time-lapse optical images displaying the sweat sampling in the microfluidics after iontophoresis. Scale bar, 2 mm. (**H**) Optical image of the 3D-printed e^3^-skin conformally adhering to the skin. Scale bar, 1 cm. (**I** and **J**) Responses of the temperature, glucose, alcohol, and pH sensors in selectivity test (I) and mechanical bending test (J). For the selectivity test, 50 μM glucose, 5 mM alcohol, and 50 μM UA were sequentially added into PBS for analysis. For the mechanical bending test, raw sweat was analyzed, while the e^3^-skin was strained at 5- and 2-cm bending radius. (**K**) Representative live (green)/dead (red) images of HDF seeded on the control (top) and an e^3^-skin (bottom) after 1- and 7-day culture. Scale bar, 100 μm. (**L**) Quantitative analysis of cell metabolic activity over a 7-day culture period. Error bars represent the SD from five measurements.

To enable on-demand and continuous molecular monitoring, a miniaturized microfluidics with a built-in iontophoretic sweat induction module, composed of hydrogels containing carbachol (carbagel), and iontophoresis electrodes (IP cathode and IP anode], was developed to interface with the 3D-printed biochemical sensors (Fig. 4A). Autonomous and long-lasting sweat induction was realized via transdermal delivery of muscarinic agent carbachol (Fig. 4B). The sweat-sampling microfluidics was 3D-printed using an SBS ink of 25 wt % with an appropriate viscosity to achieve high printing resolution (Fig. 4C and movie S2), while the iontophoresis module was prepared by printing a pair of CNT-SBS electrodes followed by gelatin-agarose carbagels (Fig. 4, D and E). Localized sweat induction using the 3D-printed iontophoresis module was realized by delivering a very small dose of carbachol with a current ranging from 1 to 3 μA mm^−2^. The secreted sweat volume was found to be linearly correlated with the applied current/delivered drug dose (Fig. 4F). During the on-body test, the induced sweat was sampled through the microfluidics to ensure that newly secreted sweat flows through the sensing reservoir with high-temporal resolution toward real-time wearable analysis (Fig. 4G and movie S3).

The assembled 3D-printed microfluidic e^3^-skin could conformally adhere to the skin (Fig. 4H) and displayed excellent selectivity and stable sensor performance under mechanical deformations (Fig. 4, I and J, and fig. S29). The e^3^-skin’s high biocompatibility and low cytotoxicity were validated by culturing human dermal fibroblasts (HDFs) and normal human epidermal keratinocyte (NHEK) cells using a commercial live/dead kit and PrestoBlue assay, as represented in Fig. 4 (K and L) and fig. S30. The viability of HDF and NHEK cells remained >95%, and their metabolic activities consistently increased during the 7-day culture.

### 3D-printed wearable energy system for the e^3^-skin

**Fig. 5.**
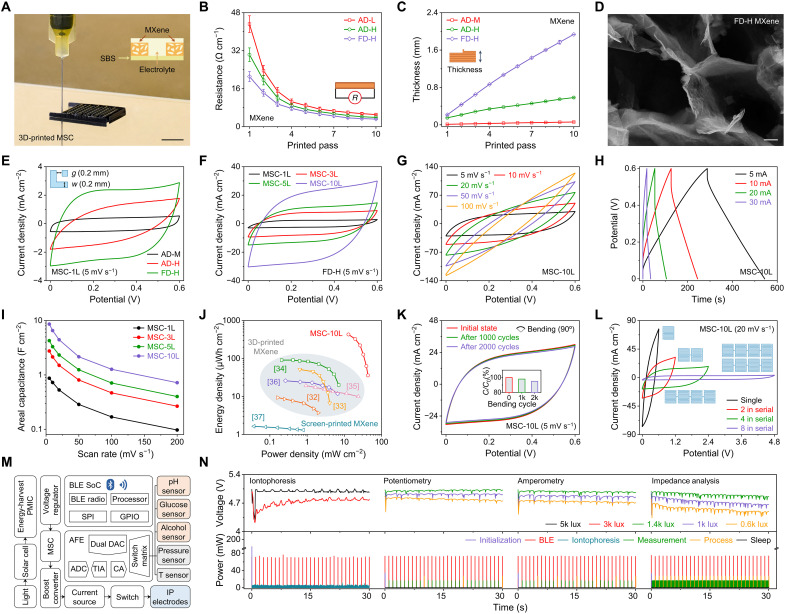
Characterization of the 3D-printed wearable energy system for the e^3^-skin. (**A**) Optical image of the SSE-based 3D-printed interdigital MXene MSC. Scale bar, 1 cm. (**B** and **C**) Resistance (B) and thickness (C) of the different MXene electrodes with the number of printed layers (*p*). Error bars represent the SD from five measurements. AD, air-dried MXene; FD, freeze-dried MXene; M, moderate concentration; H, high concentration. (**D**) SEM image of the porous FD-H MXene. Scale bar, 1 μm. (**E** to **G**) CV curves of different MXene MSCs (E), FD-H MXene MSC-*p*L with varying printed layers (F), and MSC-10L with varying scan rates (G). (**H**) GCD profiles of MSC-10L with different charging-discharging currents. (**I**) Areal capacitance plotted as a function of scan rate for MSCs with varying printed layers. (**J**) Ragone plot comparison of this work (MSC-10L) with previously reported 3D-printed and screen-printed MXene MSCs. (**K**) CV curves of the MSC-10 L for 2000 bending cycles. Inset, the corresponding capacitance retention plot. (**L**) CV curves of the MSCs connected in series to achieve the desired working potential. (**M**) System-level block diagram of the e^3^-skin with the wearable energy system. (**N**) Charging-discharging curves of the serially connected MSCs (top) and power consumption profiles (bottom) of various operation modes under different illumination conditions.

Wearable systems with miniaturized energy-harvesting and storage devices are highly desired to promote sustainability and untethered battery-free operations (*34*–*37*). Here, we designed high-performance 3D-printed MXene MSCs that can interface with a solar cell to power the e^3^-skin. A highly concentrated MXene ink (MX-H; 120 mg ml^−1^) was used to print the 3D freestanding interdigital MSC (Fig. 5A, fig. S31, and movie S4). The resistance and thickness can be readily tuned by adjusting the number of printed layers and by subjecting them to different posttreatments [i.e., air-drying (AD) and freeze-drying (FD)] (Fig. 5, B and C). Compared to AD posttreatment, the FD MX-H exhibits superior electrochemical performance owing to the highly porous structures with substantially enhanced active surface area and reduced impedance (Fig. 5D and fig. S32). Using this approach, a variety of complex 3D architectures can also be stably printed and well-preserved (fig. S33 and movie S5).

The charge-storage performance of the MXene MSC was evaluated after printing a layer of poly(vinyl alcohol) (PVA)–sulfuric acid (H_2_SO_4_) gel electrolyte on top of the interdigital MXene electrodes (figs. S34 and S35). Such gel electrolyte is commonly used in wearable energy devices (*38*). Here, the encapsulated gel electrolyte used in this work demonstrated high biocompatibility (fig. S36) and was assembled without direct contact with the skin to avoid any potential irritation. As illustrated in Fig. 5E, proportional to the area enclosed in the cyclic voltammogram (CV), the areal capacitance of FD-H MXene MSC was outstanding compared to other AD MXene MSC. For the detailed investment of FD-H MXene MSC, the electrochemical performance and ion transport property were further improved with the increased electrode dimensions (number of interdigital pairs, length, and number of printed layers) and the reduced electrode gaps (Fig. 5F and figs. S37 and S38). The CV curves and galvanostatic charge-discharge (GCD) profiles of the FD MSCs showed an electric double-layer capacitive and high-rate behavior (Fig. 5, G and H). In particular, MSC with 10 printed layers (MSC-10L) exhibited an extremely high areal capacitance of 8.61 F cm^−2^ at a scan rate of 5 mV s^−1^ and was able to discharge at a current of up to 30 mA that is adequate for practical wearable applications and for initiating wireless Bluetooth communications (Fig. 5I). Compared to previously reported printed MXene MSCs (*39*–*44*), this MSC-10L showed a superior energy density that reached as high as 12.91 μWh cm^−2^ at a power density of 439.35 mW cm^−2^ and 43.18 μWh cm^−2^ at the highest power density of 35.99 mW cm^−2^ (Fig. 5J). Moreover, it displayed robust mechanical and satisfied electrochemical cycling stability with capacitance retention of 95 and 87% after 2000 bending and scanning cycles, respectively (Fig. 5K and fig. S39). This can be attributed to the excellent mechanical properties and strong adhesion between the MXene nanosheets (*45*, *46*). To achieve desired working potential and capacitance, the 3D-printed MSCs can be serially connected, where a voltage output of 4.8 V was achieved by connecting eight MSCs in series to potentially power our wearable sensor with Bluetooth transmission (Fig. 5L).

To demonstrate the full potential of SSE-based 3D-printing technology in wearable sensing, the disposable 3D-printed e^3^-skin (consisting of biophysical temperature and pulse sensors, biochemical sensors, iontophoresis-integrated microfluidics, and MSC) was integrated with a reusable flexible printed circuit board (FPCB) coupled with a commercial solar cell that is equipped for energy harvesting, signal processing, and wireless communication (Fig. 5M). The charging-discharging profiles of the MXene MSCs in the e^3^-skin during various operations (e.g., iontophoresis for sweat induction, potentiometry for pH sensing, amperometry for glucose and alcohol sensing, and resistance measurements for biophysical monitoring) under different illumination conditions and their corresponding power consumption are demonstrated in Fig. 5N. When illuminated, the solar cell charged the MXene MSCs to ~5 V before initializing the system and was able to continuously power the wearable device to perform multiplexed electrochemical measurements, signal processing, and data transmission via Bluetooth low energy (BLE) (fig. S40).

### Evaluation of the fully integrated e^3^-skin–wearable system in human subjects

**Fig. 6.**
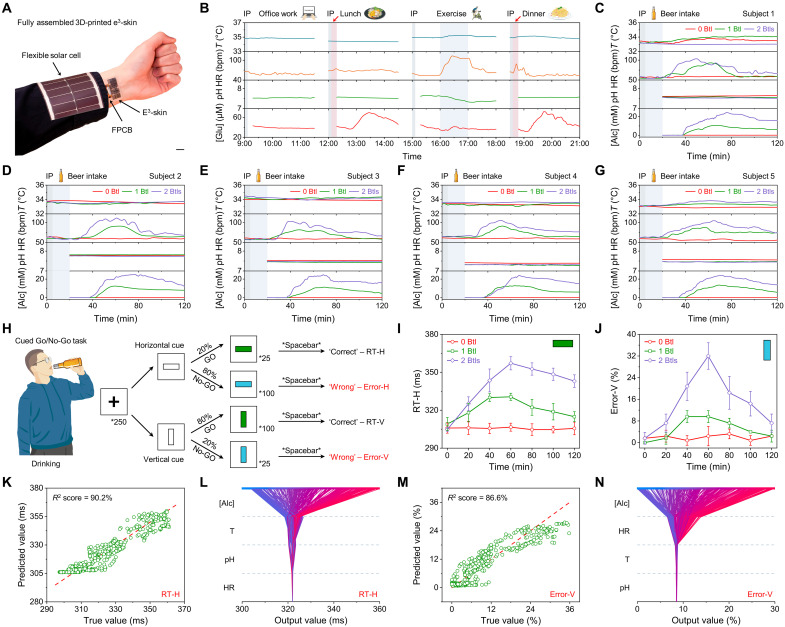
On-body evaluation using e^3^-skin for real-time health surveillance and machine learning–powered health condition prediction. (**A**) Optical image of the wearable e^3^-skin assembled with a reusable flexible printed circuit board (FPCB) and a flexible solar cell. Scale bar, 1 cm. (**B**) Full-day physiochemical surveillance of a subject while performing various activities. HR, heart rate; bpm, beats per minute. (**C** to **G**) Multiplexed multimodal physiological monitoring of five subjects after consuming alcoholic beverage with different doses. (**H**) Workflow of the cued Go/No-Go task for quantitative measurements of the deviation in RT and commission errors (%) for the DI for inhibitory control due to the influence of alcohol. (**I** and **J**) Mean RT to Go targets with horizontal cues (RT-H) (I) and commission errors (%) to No-Go targets with vertical cues (Error-V) (J) for five subjects under three alcohol conditions. Error bars represent the SD from five measurements. (**K** to **N**) The actual performance versus ML-predicted RT-H (K) and Error-V (M), and the corresponding SHAP decision plot explaining how each regression model arrives at final task performance outcome of RT-H (L) and Error-V (N).

The capability of the e^3^-skin to perform continuous multimodal sensing offers powerful surveillance and early prediction capabilities to assess an individual’s state of health in real time (Fig. 6A). On-body evaluation of the e^3^-skin was first conducted on three healthy subjects in a glucose tolerance test to simultaneously monitor the vital signs and glucose level (figs. S41 and S42). During the entire period, all vital signs remained relatively stable, while glucose consistently increased rapidly after 40 min, peaked at ~60 min, and recovered to baseline after 80 min. Multimodal monitoring was then conducted continuously over a 12-hour period in a subject involving various daily activities (Fig. 6B). Stable sensor responses were obtained when the subject was in a fasting state and performing sedentary work in the morning. After food intake (i.e., lunch and dinner), glucose level increased after ~30 to 50 min and peaked at ~75 to 90 min before it gradually decreased. Elevated heart rate and temperature were also observed when the subject was performing strenuous cycling exercise.

### Artificial intelligence–powered 3D-printed e^3^-skin for behavioral response assessment

The prevalence of drinking and the detrimental consequences could substantially influence our cardiac/metabolic activities as well as cognitive and behavioral impairments that ensue (*47*–*50*). It is well documented that even at low to moderate dosages of alcohol, our reaction time (RT), coordination, vigilance, judgment, and ability to self-control can be impaired. Although the delayed behavioral response caused by alcohol intake could have major consequences, measuring alcohol levels alone is often insufficient to determine an individual’s behavioral response given the intrapersonal variations in alcohol tolerance. Alternative approaches such as behavior questionnaires or cognitive tasks could provide accurate assessment but are impractical to implement because of the impaired self-awareness after alcohol intake. We demonstrate here in a pilot study that real-time collected multimodal data (including both alcohol and vital signs) by the e^3^-skin from human subjects, when coupled with machine learning algorithms, could accurately predict an individual’s behavioral response, as indicated by their RT and the degree of impairment (DI) for inhibitory control.

The physiological responses from five healthy subjects after zero, one, and two bottles of beer intake [355 ml, 5% alcohol by volume (ABV)] are illustrated in Fig. 6 (C to G). It was observed that both heart rate and sweat alcohol concentration elevated with increasing dosage of alcohol, while changes in temperature and pH remained subtle. In contrast to heart rate, which increased instantaneously after drinking, sweat alcohol level gradually increased over time, following a similar trend to its blood counterpart. The on-body sweat analysis was validated by in vivo analysis of the sensor with the blood alcohol content (BAC) measured using a commercial breathalyzer (fig. S43). The cognitive and behavioral responses of the subjects were further evaluated by performing a cued Go/No-Go task over a 2-hour period with 20-min intervals after alcohol intake (Fig. 6H and Materials and Methods), which quantitatively measured the deviation caused by alcohol-induced delay in the RT and DI. It is observed that the RT with horizontal cues (RT-H) as well as the number of commission errors with vertical cues (Error-V), which is associated with DI for inhibitory control (*51*, *52*), increased with higher dosages of alcohol on all subjects (Fig. 6, I and J, and fig. S44).

To predict the influence of alcohol on an individual’s performance, machine learning was performed to infer the task performance outcomes from all the physiological data acquired by the e^3^-skin. Both the RT and commission error (%) were studied using a ridge regression model. The features of the raw data were extracted with moving average, and a train-test split was performed to evaluate the model. The regression model was able to provide high-accuracy prediction for both the RT and commission error (%) in the study, with an accuracy of 90.2 and 86.6%, respectively, in *R*^2^ score (Fig. 6, K and M). The importance of each feature in the prediction method, as well as the decision-making process of the machine learning model, was evaluated using Shapley additive explanation (SHAP) values (Fig. 6, L and N, and fig. S45) (*53*). The results indicate that sweat alcohol contributed to the most substantial role in predicting the RT and heart rate was required to supplement sweat alcohol measurements for more accurate prediction of DI.

## DISCUSSION

The proposed SSE-based 3D printing serves as an elegant solution for the development of sustainable wearable multimodal health monitoring devices that traditionally can only be prepared with a series of complex fabrication processes. The all-3D–printed e^3^-skin features multimodal sensing of various physicochemical biomarkers with untethered battery-free operation capabilities. Extrudable inks with suitable rheological performance based on different materials (e.g., nanomaterials, polymers, hydrogels, and other composites) were custom-designed and optimized to achieve specific device functionality. Such SSE-based 3D printing enabled efficient maskless patterning of both 2D and 3D architectures, which can fulfill all fabrication requirements for an integrated wearable device. Furthermore, we introduced a general phase elimination strategy to create highly porous microstructures critical for enhancing the devices’ performance in practical applications.

The judiciously designed all-3D–printed e^3^-skin was capable of simultaneously monitoring glucose, alcohol, and pH in sweat along with heart rate and temperature and was equipped with an iontophoretic module for localized on-demand sweat induction, a microfluidic channel for efficient sweat sampling, and an MSC as energy storage module that can be interfaced with an energy-harvesting device for battery-free sustainable operation. With the multimodal tracking capability, more comprehensive information of our body responses can be extracted and interpreted. We demonstrated its practical use for real-time health surveillance while performing regular activities throughout the day. In addition, the information collected by the e^3^-skin and analyzed with machine learning algorithms was able to provide useful knowledge for practical personalized health assessment. For example, we were able to predict with over 90% accuracy the degree of behavioral impairments (i.e., RT and inhibitory control) of an individual after the consumption of alcohol. We envision that such wearables with multimodal sensing capabilities that can be prototyped using the presented simple and low-cost 3D-printing technology will find its use for both regular remote health surveillance and clinical applications.

## MATERIALS AND METHODS

### Materials and reagents

SBS, PEG, BSA, chitosan, carbachol, PU, PANI (emeraldine base), gelatin, GOx (from *Aspergillus niger*), and AOx (from *Pichia pastoris*, 10 to 40 U/mg of protein) were purchased from Sigma-Aldrich. Graphite flake, agarose, PVA, and UA were purchased from Alfa Aesar. Toluene, dextrose (d-glucose), sodium chloride (NaCl), hydrogen peroxide (H_2_O_2_) [30% (w/v)], sulfuric acid (H_2_SO_4_), dimethylformamide (DMF), potassium chloride (KCl), 10× phosphate-buffered saline (PBS), ethanol, lithium fluoride (LiF), and iron (III) ferrocyanide (Fe_4_[Fe(CN)_6_]_3_) were purchased from Fisher Scientific. Iron (III) chloride (FeCl_3_) and tetrahydrofuran (THF) were purchased from Thermo Fisher Scientific. Potassium ferricyanide (III) (K_3_Fe(CN)_6_) and agarose were purchased from Acros Organics. Hydrochloric acid (HCl) was purchased from VWR. Silver (Ag) paint was purchased from SPI Supplies. MAX phase powder was purchased from Jilin 11 Technology. Multiwalled CNT was purchased from Beijing Boyu Co. (China). CB was purchased from MTI Corp. PDMS (SYLGARD 184) was purchased from Dow Corning.

### Preparation of customizable inks

#### MXene ink

MXene nanosheets were synthesized using the minimally intensive layer delamination method (fig. S4) (*54*). Two grams of LiF was dissolved in 40 ml of 9 M HCl, and 1 g of Ti_3_AlC_2_ MAX phase powder was gradually added into the etchant under continuous stirring at room temperature. The mixture proceeded to react for 48 hours at 40°C. Once the reaction was complete, the acidic mixture was washed multiple times with deionized water by centrifugation at 5000 rpm for 5 min each cycle until the pH turned neutral. The dark green supernatant was discarded, and 20 ml of deionized water was added to the sediment. This was followed by vigorous shaking using a vortex machine for 1 hour. The dispersion was then centrifuged at 2000 rpm for 5 min, and the supernatant was collected. The solution containing exfoliated MXene nanosheet dispersion was centrifuged again at 5000 rpm to remove the residual impurities. After discarding the supernatant, the aqueous “clay-like” MXene sediment was collected and mixed homogeneously using a planetary centrifugal (Thinky, AR-100) for 5 min. The MXene ink at this point owned a concentration of ~60 mg ml^−1^, which was used to print the MXene interconnects and biophysical sensors. The desired concentration of the ink can be tuned by either diluting it with water or subjected to gentle evaporation in a vacuum oven. For printing of MXene MSCs and other 3D architectures, a highly concentrated ink of ~120 mg ml^−1^ was used.

#### MX-PB ink

A solution containing 5 mM FeCl_3_, 5 mM K_3_[Fe(CN)]_6_, 100 mM KCl, and 100 mM HCl was added dropwise into MXene dispersion (1 mg ml^−1^) at a volume ratio of 1:1 under continuous stirring. The mixture was then bath-sonicated for 30 min to speed up the reduction process of PB nanoparticles (note S1). For the washing process of MX-PB ink, it was performed by replacing the supernatant after each centrifugation with deionized water for three times. The desired concentrations of MX-PB (1 and 10 mg ml^−1^) for the mediator of the biochemical sensors were obtained by adding the appropriate amount of deionized water.

#### SBS and SBS composite inks

SBS ink (25 wt %) was prepared by dissolving SBS in toluene by ultrasonication. To formulate CNT-SBS, graphite-SBS, and CB-SBS inks, a specified amount of the respective fillers was homogeneously mixed into the SBS ink using a planetary centrifugal for 5 min. To develop CNT-SBS-PEG ink for the preparation of WEs, 7 wt % PEG and 9 wt % CNT were homogeneously mixed into the SBS ink. On the basis of the phase separation strategy, by immersing the as-printed electrode in the deionized water at room temperature for 1 hour, porous CNT-SBS electrode was obtained with the complete removal of PEG due to PEG’s high solubility and fast dissolution rate (Fig. 3, B and C). Through the modulation of the inks with different CNT and PEG compositional ratios, we can further optimize porous CNT-SBS electrodes with satisfying electrochemical performance. Similarly, to obtain porous CNT-SBS-PANI electrodes, 5 wt % CNT, 5 wt % PANI, and 7 wt % PEG were homogeneously mixed into the SBS ink and PEG was subsequently dissolved in deionized water after printing (Fig. 3K).

#### CNT-PDMS ink

Multiwalled CNTs (3 wt %) (diameter, 10 to 20 nm; length, 10 to 30 μm) were dispersed with PDMS base resin through the aid of toluene at a volume ratio of 1:4. The mixture was magnetically stirred for 1 hour at room temperature to ensure the homogeneous dispersion of CNTs. Meanwhile, salt microparticles (size, 20 to 40 μm) were prepared by grinding the NaCl particles for 1 min with a portable electric grinder. Subsequently, the curing agent (10 wt % in PDMS base resin) and salt microparticles at varying ratios were mixed homogeneously into the dispersion with 30 min of magnetic stirring.

#### Enzyme-biopolymer ink

Chitosan (1 wt %) was dissolved in 0.1 M acetic acid, and BSA (10 mg ml^−1^) was dissolved in PBS. For glucose enzyme ink, the chitosan solution was mixed thoroughly with GOx solution (10 mg ml^−1^ in PBS) at a volume ratio of 2:1. For the alcohol enzyme ink, AOx was used as received without modification. Then, the chitosan and BSA solutions were mixed thoroughly with AOx at a volume ratio of 1:1:8.

#### PU diffusion–limiting membrane ink

A THF solution containing 2 wt % DMF was prepared followed by dissolving 3 wt % PU by ultrasonication.

#### Agarose-gelatin carbagel

Agarose (2 wt %) and gelatin (5 wt %) were added into deionized water and heated at 250°C under continuous stirring until the solution turned homogeneous. The mixture was then cooled down to 165°C, and 1 wt % carbachol or KCl was then added into the mixture to develop carbagel for printing on the anode and cathode, respectively.

#### PVA-H_2_SO_4_ gel electrolyte

PVA (10 wt %) was dissolved in 10 ml of deionized water and mixed with 1 g of H_2_SO_4_. The mixture was heated up to 90°C under vigorous stirring for 1 hour until the solution became clear.

### Material characterization

The rheological properties of the customizable inks were analyzed using a rheometer (Anton Paar, MCR 302). The surface morphologies and structures of the printed electrodes and devices were studied by optical microscopy (ZEISS) and scanning electron microscopy (SEM; ZEISS 1550VP FESEM). The SEM was equipped with an energy-dispersive x-ray spectroscopy analyzer (Oxford X-Max SDD) to determine the element composition of as-printed MXene and MX-PB. The microstructures and material properties were further characterized using x-ray diffraction (Rigaku, Miniflex II), TEM (JEOL, 2100F), and Raman spectroscopy (WITec, CRM200). The lateral size and thickness of the MXene nanosheets were characterized using atomic force microscopy (Asylum Research Cypher S). Contact angle measurements of the various printed MXene were acquired using a goniometer (ramé-hart).

### SEE and assembly of the e^3^-skin

The SSE-based 3D-printing sequence and assembly of the e^3^-skin is schematically presented in fig. S2. The SBS substrate was printed on glass slide, and all electrodes and devices were printed in sequence on the SBS substrate (unless specified otherwise) using a three-axis robotic deposition stage (Aerotech). The printing paths were designed using AutoCAD software and translated into G-code using a custom Python script. The inks were loaded into 15-ml syringe barrels and fitted with appropriate nozzles ranging in sizes from 90 to 580 μm (fig. S1). The extrusion of ink was controlled by applying air pressure using a benchtop fluid dispenser (EFD Nordson). The printing pressure and speed were optimized for each individual ink to achieve stable extrusion. In brief, the e^3^-skin is composed of three components: the 3D-printed microfluidics, the 3D-printed biosensors, and the 3D-printed MSC. All the SBS-based inks have similar rheological performance with the toluene as the solvent, and they can be dried almost immediately within seconds because of the fast evaporation of the toluene after printing. For other aqueous inks, considering the small dimensions used in our design, the printed layer normally became dry quickly within minutes after printing. For the 3D-printed microfluidics, SBS ink was used to construct the microfluidics, which was composed of an inlet for sweat collection and a channel connecting the inlet to the sensor reservoir and routing to an outlet. After assembling the two layers, the iontophoresis carbagels were then subsequently printed at the designated openings. For the 3D-printed biosensors, first, the MXene interconnects and electrodes for the temperature and pulse sensor were printed on the SBS substrate. Then, CNT-SBS ink was used to print the WEs for the biochemical sensors, counter electrode, and iontophoresis electrodes, while CNT-SBS-PANI was printed for the pH sensor and commercial Ag paint was printed as the reference electrode. Following this step, the patch was removed from the printing bed and immersed in deionized water for 1 hour to fully dissolve PEG to induce porous structure. After the patch was completely dry, the glucose and alcohol sensors were then prepared by sequentially dispensing 3 μl of MX-PB with different desired concentrations and their corresponding enzyme biopolymer inks on the respective CNT-SBS WEs. For alcohol sensor, a PU diffusion–limiting layer was additionally printed. The disposable microfluidic sensor patches containing biosensors and carbagel-loaded electrodes, the fabricated biosensors, and the iontophoretic modules were stored in a sealed container in the refrigerator at 4°C before use. Last, 3D interdigital MXene electrodes were printed on the SBS substrate layer by layer and immediately removed from the printing bed for FD treatment in a freeze dryer (SP Scientific AdVantage 2.0 Benchtop) to induce porous architectures for improved performance. PVA-H_2_SO_4_ gel electrolyte was then printed over the electrodes, and the entire MSC connected in series was dried under the fume hood overnight at room temperature to vaporize the excess water.

### Characterization of biophysical sensors

Uniaxial compression test of the pressure sensor was performed using a force gauge (Mark-10), and the temperature sensor was characterized on a ceramic hot plate (Thermo Fisher Scientific). For in vitro characterization of the temperature sensor and pulse sensor, a parameter analyzer (Keithley 4200A-SCS) was used to record the resistance response.

### Numerical simulation for pressure sensor

Finite element analysis (FEA) was used to analyze the deformation behavior of CNT-PDMS with different surface architectures. A static nonlinear analysis under the contact condition was carried out by the open-source FEA software Code_Aster. In the simulation, each surface architecture was contacted with a rigid body and deformed elastically upon increasing contact depth. Randomly porous structure was simplified to spherical voids with identical spacing. The Young’s modulus (*E*) and Poisson’s ratio (*v*) of CNT-PDMS were set to *E*_CNT-PDMS_ = 1 MPa and *v*_CNT-PDMS_ = 0.499, respectively.

### Electrochemical characterization of biochemical sensors and MSCs

Electrochemical characterizations of the sensors and MSCs were carried out using an electrochemical workstation (CHI 660E). For the characterization of UA sensor, 0 to 200 μM of UA solutions were prepared in PBS (pH 7.4) and differential pulse voltammetry responses were measured over a potential range of 0 to 0.6 V with 4-mV step potential, 500-ms pulse period, 50-ms pulse width, and 50-mV pulse amplitude. For in vitro H_2_O_2_ and enzymatic sensor characterizations, H_2_O_2_, glucose, and alcohol solutions ranging from 0 to 5 mM, 0 to 200 μM, and 0 to 20 mM were prepared in PBS (pH 7.4), respectively. Mcllvaine’s buffers with pH values ranging from 4 to 8 were used to characterize the pH sensor. In detail, the H_2_O_2_ and enzymatic sensors were characterized amperometrically at a potential of 0 V, and the pH sensor was characterized using open circuit potential measurement. Both amperometric and potentiometric responses were set at 0.1-s sampling interval. Characterization of temperature influence on the sensors was carried out on a ceramic hot plate.

The performance of the MSCs was evaluated using CV at different scan rates ranging from 5 to 200 mV s^−1^ and GCD with different currents ranging from 0.5 to 30 mA in a voltage window of 0 to 0.6 V. Long-term cycling stability was analyzed using CV at a scan rate of 5 mV s^−1^ for 2.000 cycles. The areal capacitance was calculated using the derived equation based on the CV curve

$$C_{A}=\frac{Q}{A\cdot \text{Δ}V}=\frac{1}{k\cdot A\cdot \text{Δ}V}\;\int_{V_{1}}^{V_{2}}I(V)dV$$ (1)

where *C*_A_ is the areal capacitance, *I*(*V*) is the discharge current function, *k* is the scan rate, *A* is the effective area of the MSC, Δ*V* is the potential window during the discharging process, and *V*_1_ and *V*_2_ are the maximum and minimum voltage values, respectively.

For the Ragone plot, the device areal energy density and power density were calculated on the basis of the following equations

$$E=\frac{1}{2\times 3600}C_{A}{\left(\text{Δ}V\right)}^{2}$$ (2)

$$P=\frac{E}{\text{Δ}t}\times 3600$$ (3)

where Δ*t* is the discharging time, *E* is the energy density, and *P* is the power density.

### Evaluation of iontophoretic and microfluidic modules

Sweat was induced by applying a certain current to the iontophoretic module of the e^3^-skin for 5 min, and the total volume of sweat secreted was measured by using a sweat collector (Macroduct) for 30 min. In vitro flow tests were performed to evaluate the dynamic response of the enzymatic sensors using a syringe pump (78–01001, Thermo Fisher Scientific), where different fluids were injected into the preassembled e^3^-skin using a flow rate of 1 to 4 μl min^−1^. For the on-body flow test, a tiny droplet of red dye was predeposited in the sweat reservoir of the microfluidics and the e^3^-skin was attached to the forearm region of a subject.

### In vitro validation of glucose and alcohol sensors

The glucose and alcohol sweat sensors were validated by comparing the measurements in sweat with a commercial blood glucose meter (Care Touch) and breathalyzer (BACtrack S80 Pro), respectively. In both experiments, the subject was iontophoretically simulated on the forearm region and sweat was collected and stored in separate vials every 20 min. For glucose sensor validation, the blood glucose level of the subject was measured during a fasting state and after food intake at every 20-min intervals. For alcohol sensor validation, the BAC using a breathalyzer was measured at 10-min intervals after consuming one or two bottles of beer.

### In vitro cell studies

#### Cell lines

Normal adult HDFs (Lonza) and NHEKs (Lonza) were cultured at 37°C and under 5% CO_2_ atmosphere. Before test, cells were passaged at 70 to 80% confluency and a passage number of five to six was used for in vitro cell studies.

#### In vitro cytocompatibility studies

The e^3^-skin was washed with 70% ethanol (three times) and transferred to 24-well permeable cell culture inserts (Nunc). Next, each well was seeded with HDF and NHEK cells (1 × 10^5^ cells per well), and the inserts with e^3^-skin samples were placed in cell-seeded wells. The cells were then treated with recommended media and incubated at 37°C and under 5% CO_2_ atmosphere during the study. For control, inserts without samples were loaded in the wells.

### Electronic system design and characterization

The electronic system of the e^3^-skin consists of four main blocks: the power management block, which includes an energy-harvesting power management integrated circuit (PMIC) (BQ25504, Texas Instruments) and a voltage regulator (ADP162, Analog Devices); the data processing and wireless communication block, which includes a compact programmable system-on-chip (PSoC) BLE module (Cyble-222014, Cypress Semiconductor) and a microcontroller and BLE radio; the electrochemical instrumentation block, which includes an electrochemical front-end chip (AD5941, Analog Devices) that contains configurable amplifiers for various electrochemical measurements; and the iontophoresis sweat induction block, which includes a boost converter (TPS61096, Texas Instruments), a bipolar junction transistor (BJT) array (BCV62C, Nexperia) configured as a current mirror, and an analog switch (DG468, Vishay Intertechnology) for delivering current across the skin through the carbagel. The system’s workflow involves the PSoC BLE module acting as a data bridge between the electrochemical analog front end (AFE) and a host software, encoding and writing measurement instructions to the electrochemical AFE and transmitting the AFE’s measurement data to the host software via BLE. The power consumption of the system was characterized using a power profiler (PPK2, Nordic Semiconductor), and the charging-discharging curves of MSC were collected using an electrochemical workstation (CHI 660E).

### On-body evaluation of the e^3^-skin

The validation and evaluation of the e^3^-skin were performed using human subjects in compliance with the ethical regulations under protocols (ID 19–0892) that were approved by the Institutional Review Board at the California Institute of Technology (Caltech). Participating subjects between the ages of 22 and 40 were recruited from the Caltech campus and neighboring communities through advertisement by posted notices, word of mouth, and email distribution. All subjects gave written informed consent before participation in the study. For all human studies, subjects cleaned their skin with water and alcohol swabs before attaching the e^3^-skin on their wrists.

During on-body trials, the subjects were iontophoretically simulated and multimodal monitoring signals of temperature, heart rate, and sweat biomarkers were wirelessly transmitted via BLE. For the glucose tolerance test, subjects were given a soft drink containing 39 g of sugar and were monitored for 2 hours. For full-day evaluation, while performing daily activities, the subject was tasked to perform various activities across a 12-hour time span. For the alcohol study, zero to two bottles of beer were consumed for each subject, and they were monitored for 2 hours while performing the cued Go/No-Go task at 20-min intervals.

### Cued Go/No-Go task

Alcohol-induced delay in RT and DI for inhibitory control was measured using the cued Go/No-Go task (*44*, *45*), which was performed on a personal computer. The task comprises a sequence of events as listed in Fig. 6H. First, a fixation point (+) was displayed at the center of the screen for 800 ms, followed by a blank screen for 500 ms. An either horizontal or vertical rectangular cue with black outline was presented for one of five stimulus onset asynchronies (100, 200, 300, 400, and 500 ms) before a Go or No-Go target was displayed. The Go and No-Go targets were presented as a single green and blue hue that filled the interior of the rectangle cue, respectively. There is a total of 125 horizontal cues and 125 vertical cues. The vertical cues preceded the Go target on 80% of trials and No-Go target on 20% of trials, while the horizontal cues preceded the Go target on 20% of trials and No-Go target on 80% of trials. The subjects were instructed to respond by pressing the spacebar as quickly as possible when the Go target appeared using their index finger of the preferred hand and suppressing any action when the No-Go target appeared. The Go and No-Go targets remained visible for 1000 ms or were terminated once a response was registered. RT was calculated as the elapsed time from the Go target onset until the key was pressed, and a commission error was recorded when a key press was registered during the presentation of a No-Go target.

### Machine learning data analysis

For each model, all four features were extracted from the collected raw data of e^3^-skin: heart rate, temperature, pH, and alcohol level. We segmented the moving average data with a sampling window of 30 s to ensure that the data are semantically meaningful from each other. All biochemical data were shifted 20 min to align with physical ones given the natural sweat delay. We performed training and testing dataset split with a ratio of 8:2 before putting the data into the training model. To evaluate the model performance in the testing dataset, an *R*^2^ score was used as the evaluation metrics for the accuracy. Given the data complexity and to prevent model overfitting, we adopted a ridge regression model, whose kernel was linear regression with a 12-norm regularization. A cross-validation strategy was performed to find the best hyperparameter combination. After model training, SHAP values were used to evaluate the performance of each feature with respect to the model outcome.

## Supplementary Materials

### This PDF file includes:

Note S1 Figs. S1 to S45 Table S1 Legends for movies S1 to S5 References
