# Supplementary material for: 3D-printed epifluidic electronic skin for machine learning–powered multimodal health surveillance
Source: Sci Adv. 2023 Sep 13;9(37):eadi6492. doi: 10.1126/sciadv.adi6492 (PMC10499321; doi:10.1126/sciadv.adi6492)
Supplement: Supplementary file 1 — Note S1 Figs. S1 to S45 Table S1 Legends for movies S1 to S5 References [file sciadv.adi6492_sm.pdf]

Science Advances
AAAS

Supplementary Materials for

3D-printed epifluidic electronic skin for machine learning–powered multimodal health surveillance

Yu Song *et al.*

Corresponding author: Wei Gao, weigao@caltech.edu

Sci. Adv. **9**, eadi6492 (2023)
DOI: 10.1126/sciadv.adi6492

The PDF file includes:

Note S1
Figs. S1 to S45
Table S1
Legends for movies S1 to S5
References

Other Supplementary Material for this manuscript includes the following:

Movies S1 to S5

Note S1. ***In situ*** **synthesis of MXene-Prussian blue (MX-PB)**

Prussian blue (PB) nanoparticles is synthesized *in situ* on exfoliated MXene nanosheets when mixed in a solution containing ferric chloride and ferricyanide (**Methods**). The detailed synthetic pathway of MX-PB is depicted in **fig. S14a**. When exposed to the Fe^{3+} ions, the electrostatic-repulsive forces between the MXene nanosheets are destroyed as the positively charged metal ions spontaneously bind to the negatively charged surface of MXene (*54*). This is evident by the instantaneous aggregation of MXene which forms sediments at bottom of the dispersion. At the same time, it can also be observed that the dispersion turned blue instantaneously which suggests spontaneous formation of PB nanoparticles. This process is similar to the one-step hybridization of metallic nanoparticles with MXene using aqueous metal salt precursors (*55–57*). The formation mechanism can be explained by an *in situ* reduction-oxidation (redox) process, where the Ti on the surface of MXene serves as reductant for reducing ferric ions (*58, 59*):

$$Fe^{3+} \rightarrow Fe^{2+} \qquad (1)$$

$$Fe(CN)_6{}^{3-} \rightarrow Fe(CN)_6{}^{4-} \qquad (2)$$

A series of material characterization techniques including XRD, Raman, EDS and TEM were used to determine the formation of MX-PB (**figs. S14** and **S15**). From the XRD and Raman spectra of MX-PB, characteristic peaks originating from both MXene and PB were observed (**fig. S14B** and **S14C**). EDS mapping of MX-PB deposited on porous CNT-styrene-butadiene-styrene (CNT-SBS) revealed that all the elemental compositions of MX-PB (i.e., C, Ti, Fe and N) were uniformly distributed (**fig. S14D** and **S14E)**. The microstructure of the MX-PB was further investigated using TEM (**fig. S15)**. As can be easily observed, many PB nanoparticles were decorated uniformly on the surfaces of the MXene nanosheets. High resolution TEM images in **Fig. 3F** unveiled the presence of PB nanoparticles with measured D-spacings of 0.36 nm and 0.23 nm corresponding to (220) and (420) crystal planes of PB, respectively. The average size of the PB nanoparticles were estimated to be ~8 nm. It is noteworthy to mention that no leaching of PB nanoparticles from the MX-PB was observed after repeated centrifugation indicating strong electrostatic attraction between the MXene and PB nanoparticles.

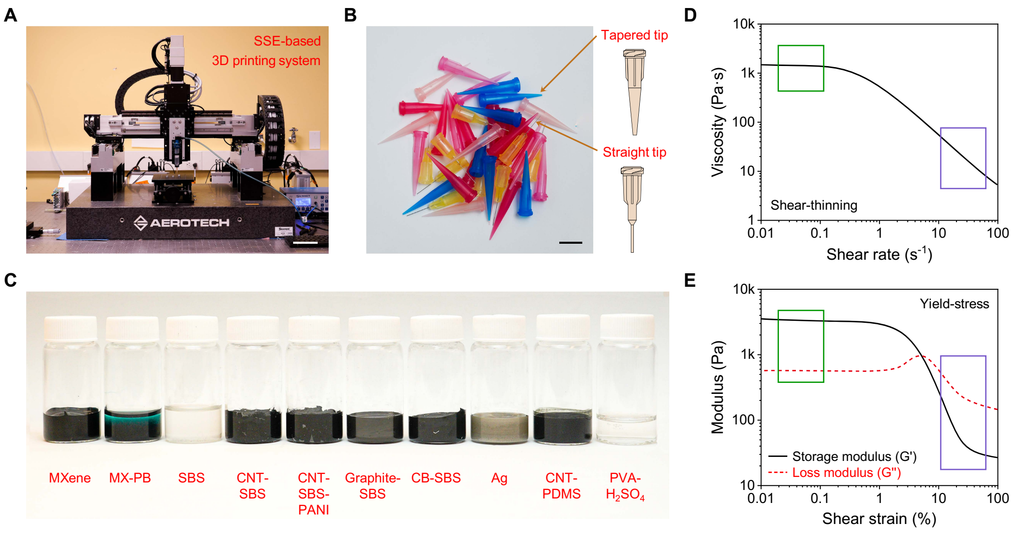

Fig. S1. 3D printing with customizable inks. (**A**) Optical image of the semi-solid extrusion (SSE)-based 3D printing system. Scale bar, 10 cm. (**B**) Optical image of the tapered tips and stainless-steel straight tips for inks with different properties. Scale bar, 1 cm. (**C**) Optical image of the customizable inks for 3D printing. (**D** and **E**) Typical plots of viscosity (D) as a function of shear rate and storage modulus (G′) and loss modulus (G″) (E) as a function of shear strain for 3D-printable inks. The green and purple boxes represent shear conditions following and during extrusion, respectively.

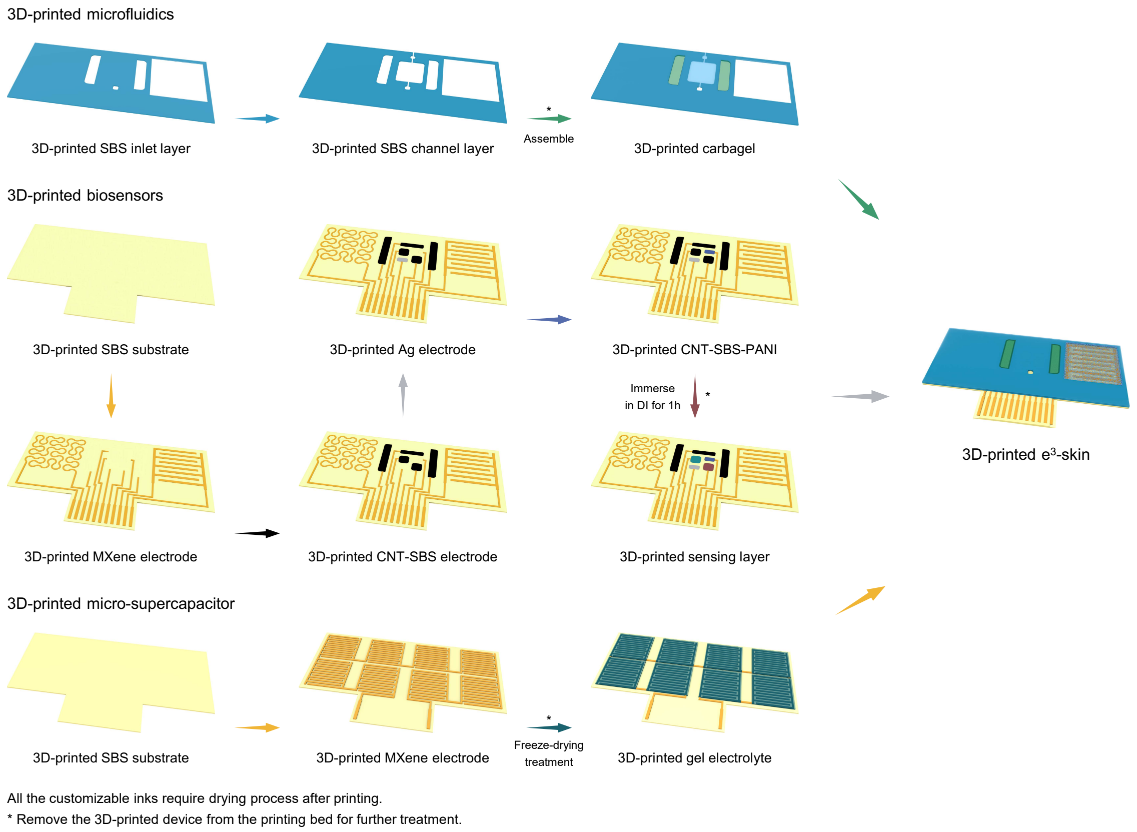

Fig. S2. Fabrication process of the 3D-printed e^3-skin. Schematic illustration of the sequential printing and assembly of the e^3-skin consisting of 3 components: 3D-printed microfluidics, 3D-printed biosensors, and 3D-printed energy storage device.

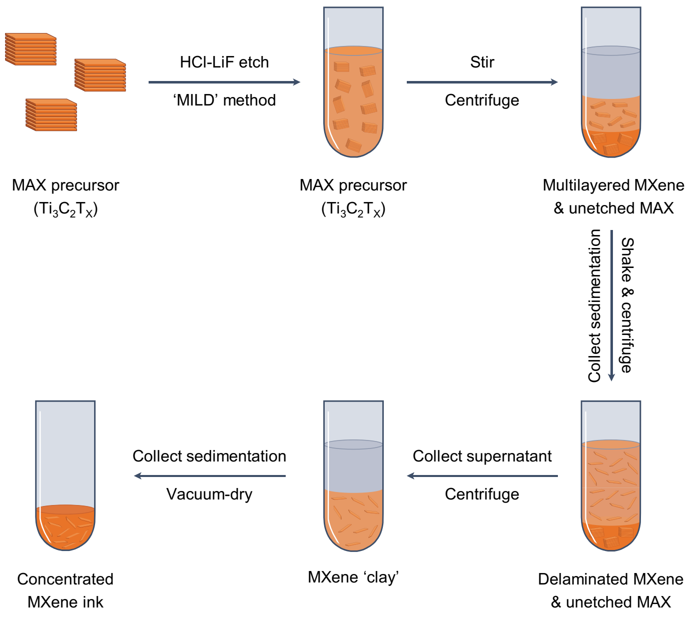

Fig. S3. Schematic illustration of the preparation process of concentrated MXene inks. MXene nanosheets were synthesized from MAX phase precursor using the minimally intensive layer delamination ('MILD') method (*60*).

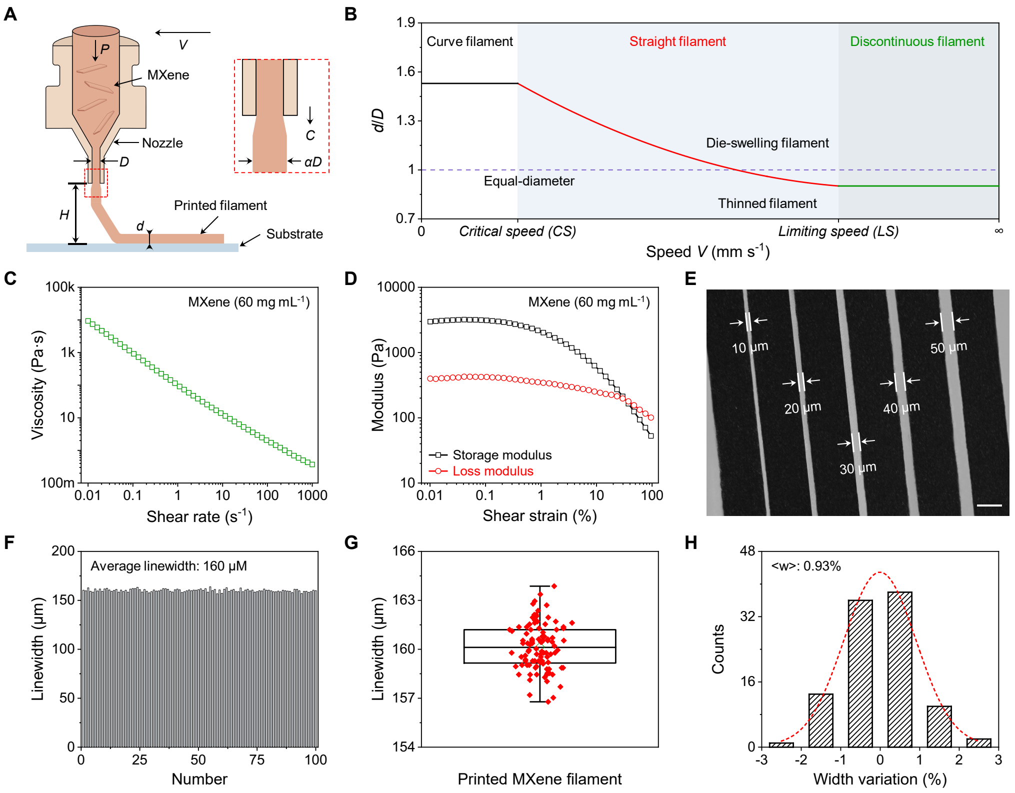

Fig. S4. Characterization of the 3D-printed filament with the MXene inks. (**A**) Schematic of the SSE-based 3D printing of MXene filament. *V*, printing speed; *P*, pressure; *D*, nozzle diameter; *H*, printing height; *d*, diameter of printed filament; *C*, extrusion speed; *α*, die swelling ratio. (**B**) Theoretical model for the diameter of the printed filament as a function of the printing speed based on the equation for volume conservation[8]:

$$d = \alpha D/\sqrt{V/C} \qquad (1)$$

For printing of straight filament, the printing speed, *V*, must be equal or greater than the critical extrusion speed, *CS*. As *V* increases, the diameter of the printed filament, *d*, decreases and the filament becomes thinner. When *V* exceeds the limiting speed, *LS*, the filament cannot be conformally deposited on the substrate resulting in discontinuity. (**C** and **D**) Viscosity as a function of shear rate (C) and storage modulus and loss modulus as a function of shear strain (D) of the MXene ink (60 mg mL^{-1}). (**E**) Microscopic image of the printed MXene filaments with gaps from 10 μm to 50 μm. Scale bar, 100 μm. (**F** to **H**) Linewidth distribution (F and G) and uniformity (H) of the 100 printed MXene filaments with an ultrahigh spatial uniformity of 0.93%.

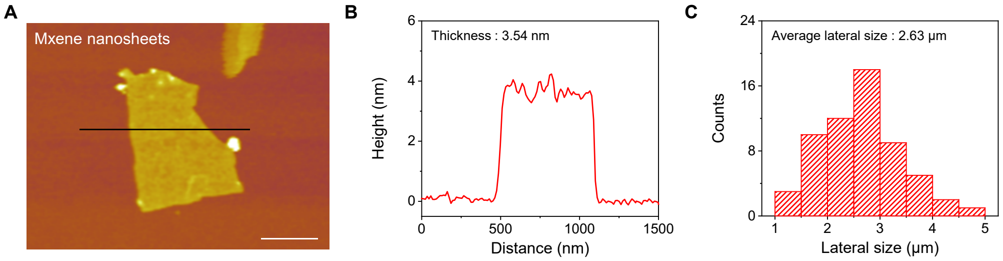

Fig. S5. Characterization of the MXene nanosheets. (**A** and **B**) Atomic force microscopy (AFM) image of MXene nanosheet (A) and its corresponding height profile (B). Scale bar, 500 nm. (**C**) The distribution of lateral size of 60 sampled MXene nanosheets.

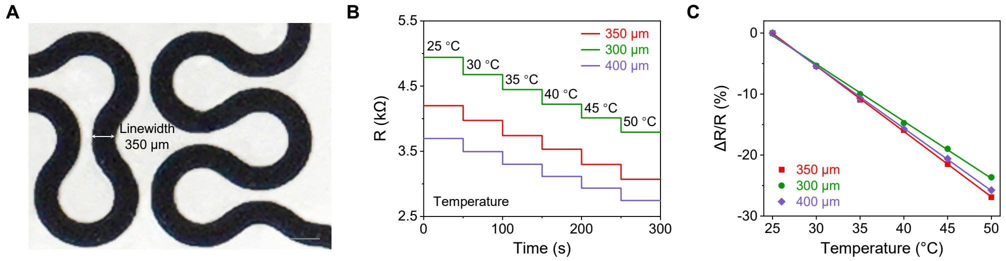

Fig. S6. Characterization of 3D-printed temperature sensors with different linewidth. (**A**) Optical image showing the linewidth of the temperature sensor in our e3-skin. Scale bar, 500 μm. (**B** and **C**) Dynamic response of the MXene-based temperature sensors with different linewidth under varying temperature (B) and the calibration plot within physiological temperature range (C).

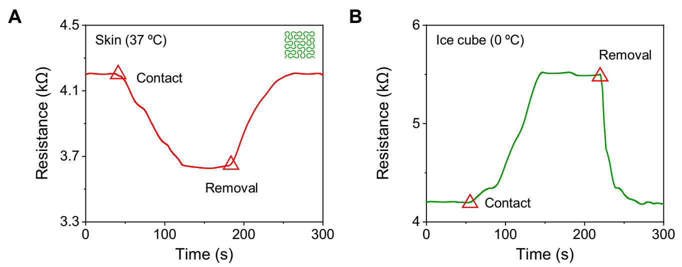

Fig. S7. Dynamic response of the MXene temperature sensor. (**A** and **B**) Response time of a MXene temperature sensor upon contact with and removal from the human skin (A) and an ice cube (B).

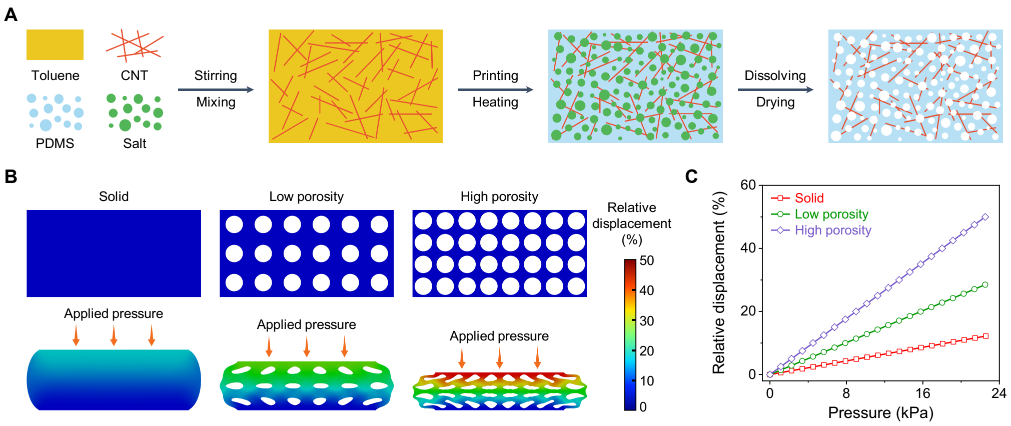

Fig. S8. 3D-printed porous CNT-PDMS. (**A**) Schematic illustration of the preparation of 3D-printed porous CNT-PDMS followed by selective phase elimination process. (**B**) Finite element method (FEM) analysis of the relative displacement of the CNT-PDMS under the same applied pressure with no porosity, low porosity, and high porosity. (**C**) FEM analysis of the relative displacement of the CNT-PDMS with different porosities under the applied pressure. PDMS, polydimethylsiloxane.

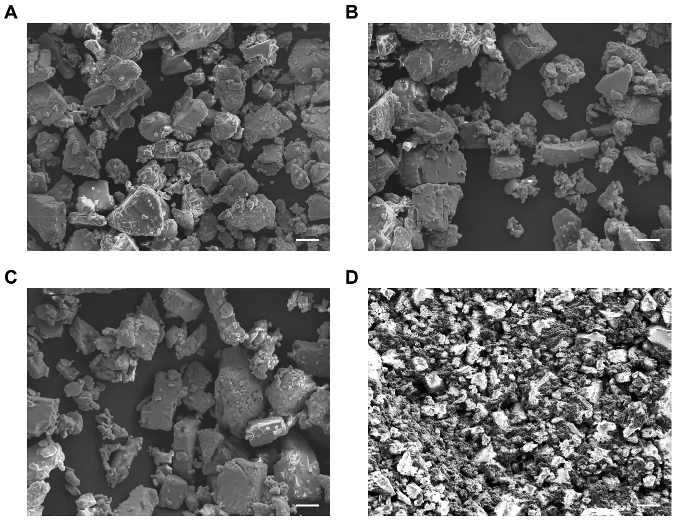

Fig. S9. Characterization of the salt microparticles. (**A** to **C**) SEM images of salt microparticles from three batches showing the constant size with 20-40 μm. Scale bars, 20 μm. (**D**) SEM image of 3D-printed CNT-PDMS before the removal of salt microparticles. Scale bar, 100 μm.

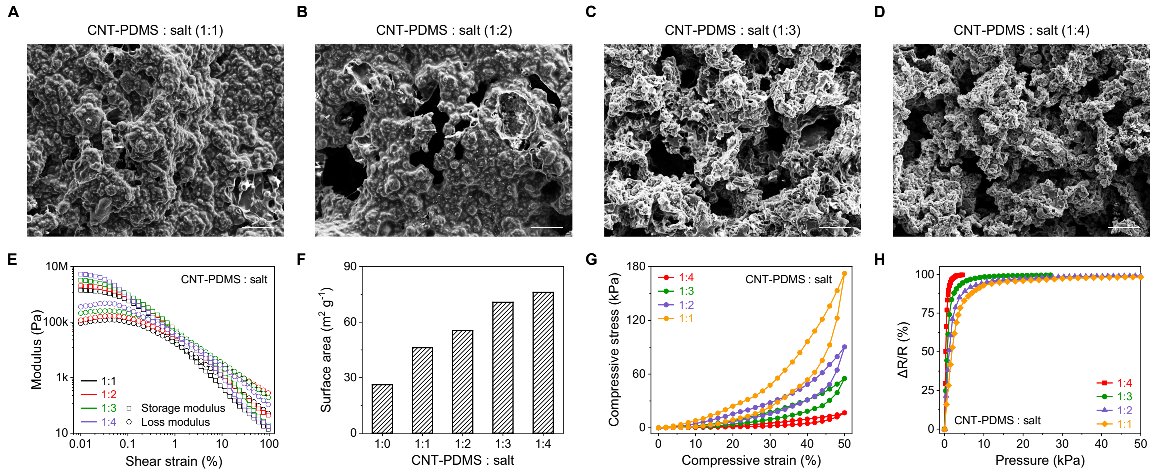

Fig. S10. Characterization and optimization of 3D-printed CNT-PDMS. (**A** to **D**) SEM images of the 3D-printed porous CNT-PDMS using a ratio of CNT-PDMS and salt of 1:1 (A), 1:2 (B), 1:3 (C) and 1:4 (D). Scale bars, 50 µm. (**E**) Storage and loss modulus of the CNT-PDMS-salt inks with varying compositional ratios as a function of shear strain. (**F** and **G**) Surface area (F) and the stress-strain curves (G) of the 3D-printed porous CNT-PDMS with varying compositional ratios. (**H**) Resistive responses of pressure sensors with varying compositional ratios under applied pressure.

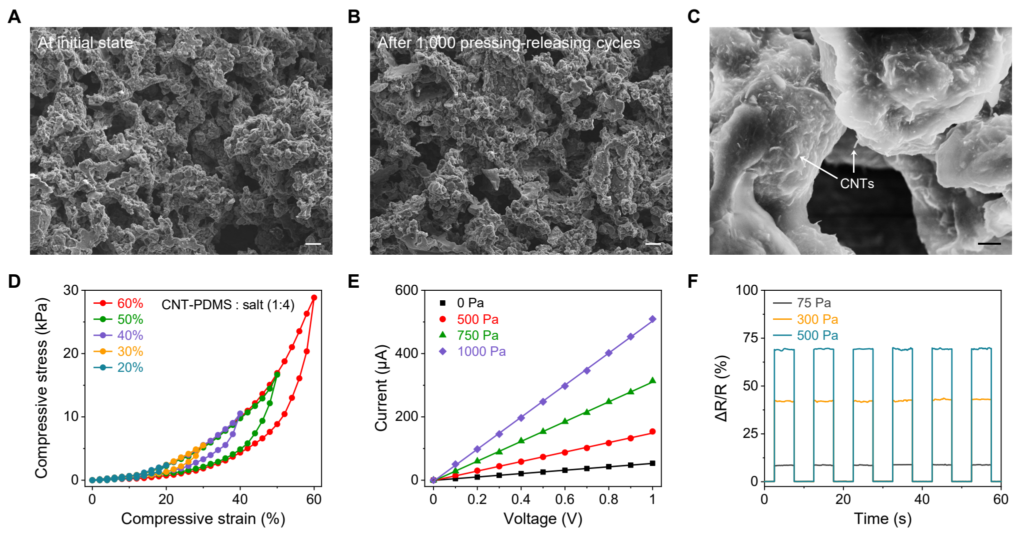

Fig. S11. Structural and mechanical characterization of the optimized porous CNT-PDMS. (**A** and **B**) SEM images of the 3D-printed porous CNT-PDMS at the initial state (A) and after 1,000 pressing-releasing cycles (B). Scale bars, 20 µm. (**C**) Magnified SEM image of porous CNT-PDMS revealing the uniform distribution of the embedded CNTs. Scale bar, 1 µm. (**D**) Sequential loading-unloading cycles of CNT-PDMS under different strains from 20% to 60%. (**E**) Current-voltage (I-V) curves of the pressure sensor under different pressure loads. (**F**) Repetitive responses of the pressure sensor under small pressure loads.

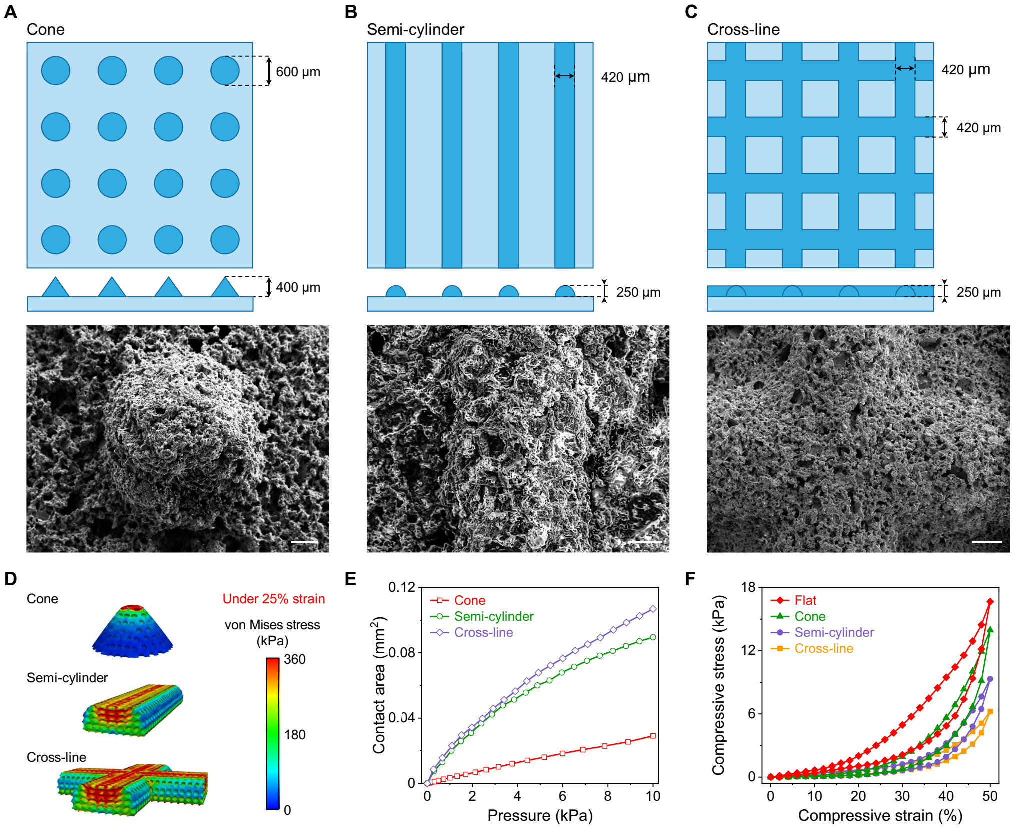

Fig. S12. 3D-printed pressure sensors with different surface architectures. (**A** to **C**) Top and cross-sectional schematic and SEM images of the 3D-printed CNT-PDMS with cone (A), semi-cylinder (B), and cross-line (C) architectures. Scale bars, 100 μm. (**D**) FEM analysis of the von Mises stress field of different architectures under 25% strain. (**E**) FEM analysis of the contact area of the 3D-printed pressure sensors with different architectures under applied pressure. (**F**) Stress-strain curves of the 3D-printed pressure sensors under maximum strain of 50%.

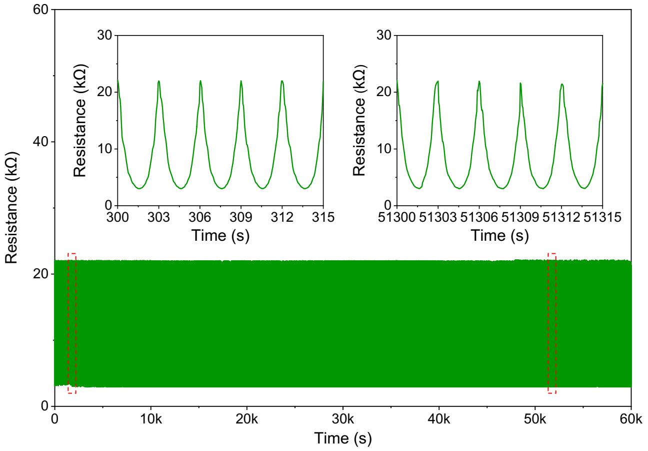

Fig. S13. Long-term stability of the piezoresistive pressure sensor. Repetitive pressure-loading test involving 20,000 pressing-releasing cycles. Insets, Dynamic responses of the pressure sensor during the 100-105 and 17100-17105 pressing-releasing cycles.

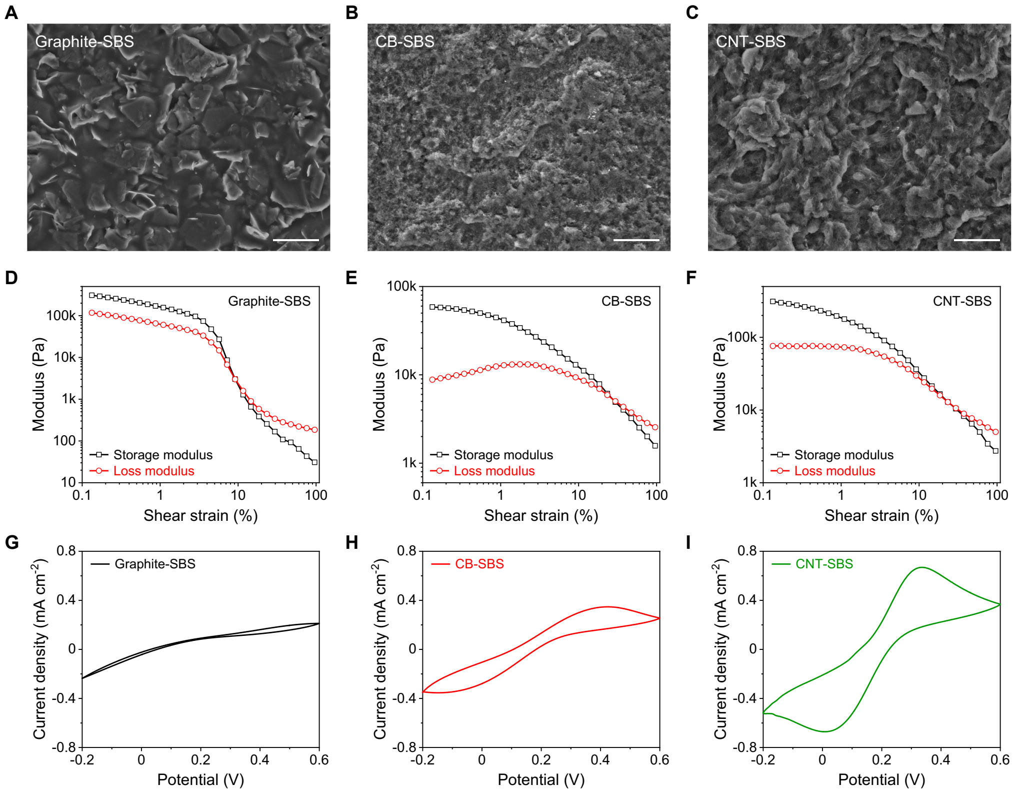

Fig. S14. Characterization of 3D-printed carbon-based electrodes. (**A** to **C**) SEM images of graphite-SBS (A), carbon black (CB)-SBS (B), and CNT-SBS (C). Scale bars, 10 µm. (**D** to **F**) Storage and loss modulus of the graphite-SBS (D), CB-SBS (E), and CNT-SBS (F) as a function of the shear strain. (**G** to **I**) Cyclic voltammetry (CV) curves of a 3D-printed graphite-SBS electrode (G), a 3D-printed CB-SBS electrode (H), and a 3D-printed CNT-SBS electrode (I) in a solution containing 5 mM $[Fe(CN)_6]^{3-}$ and 0.1 M KCl at the scan rate of 50 mV s^{-1}.

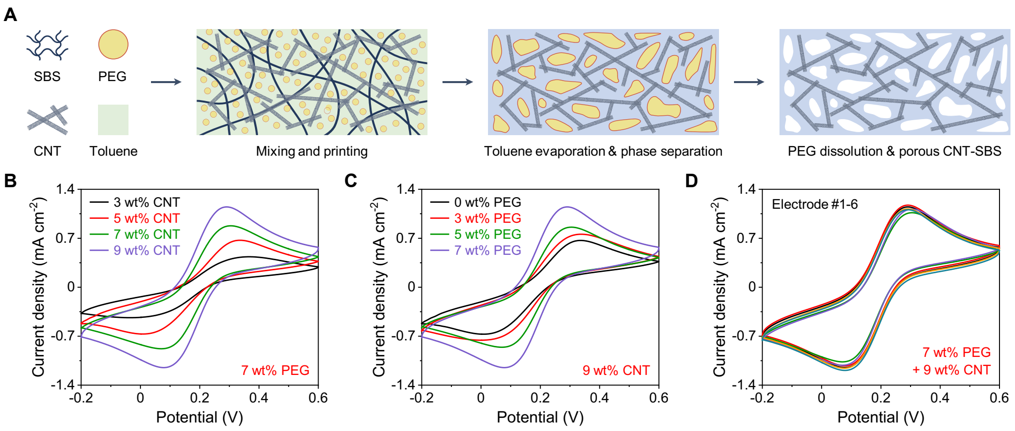

Fig. S15. Optimization of the 3D-printed porous CNT-SBS electrode. (**A**) Schematic illustration of the preparation of 3D-printed porous CNT-SBS electrode by selective phase elimination. (**B** and **C**) CV curves of a 3D-printed CNT-SBS electrode with different CNT compositional ratios (B) and different PEG compositional ratios (C) in a solution containing 5 mM $[Fe(CN)_6]^{3-}$ and 0.1 M KCl at the scan rate of 50 mV s^{-1}. (**D**) CV curves of 6 CNT-SBS electrodes in a solution containing 5 mM $[Fe(CN)_6]^{3-}$ and 0.1 M KCl at the scan rate of 50 mV s^{-1}.

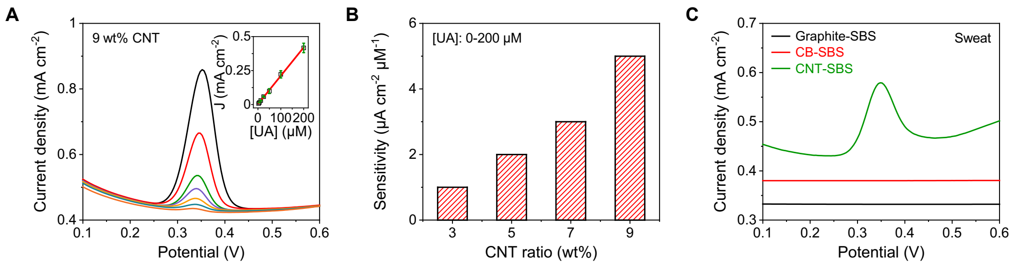

Fig. S16. Electrochemical characterization of the 3D-printed CNT-SBS electrode for uric acid (UA) detection. (**A**) Differential pulse voltammetry (DPV) curves of 3D-printed CNT-SBS-based UA sensor using 9 wt% CNT in 0–200 μM UA. Inset, the corresponding calibration plot. J, peak height current density. Error bars represent the s.d. from 5 sensors. (**B**) Sensitivity of the 3D-printed CNT-SBS-based UA sensors with different CNT compositional ratios. (**C**) DPV curves obtained in raw sweat samples using a graphite-SBS electrode, a CB-SBS electrode, and a CNT-SBS electrode.

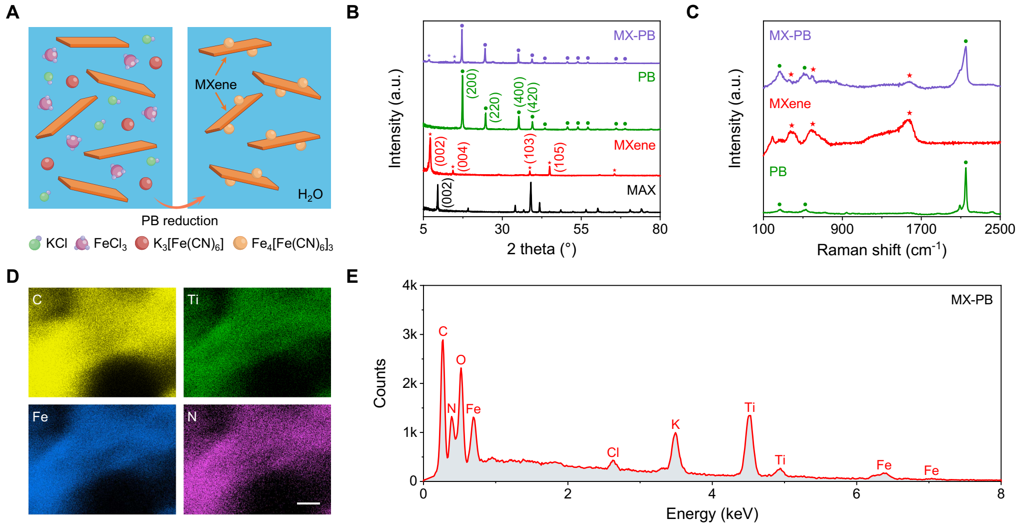

Fig. S17. Characterization and validation of *in situ* reduction of MX-PB. (**A**) Schematic illustration of the synthesis of MX-PB. (**B**) X-ray diffraction (XRD) pattern of MAX powder, MXene nanosheets, PB nanoparticles, and MX-PB. (**C**) Raman spectra of the PB nanoparticles, MXene nanosheets, and MX-PB. (**D** and **E**) Energy-dispersive X-ray spectroscopy (EDS) elemental mapping images (D) and its corresponding spectrum of MX-PB (E). Scale bar, 1 μm.

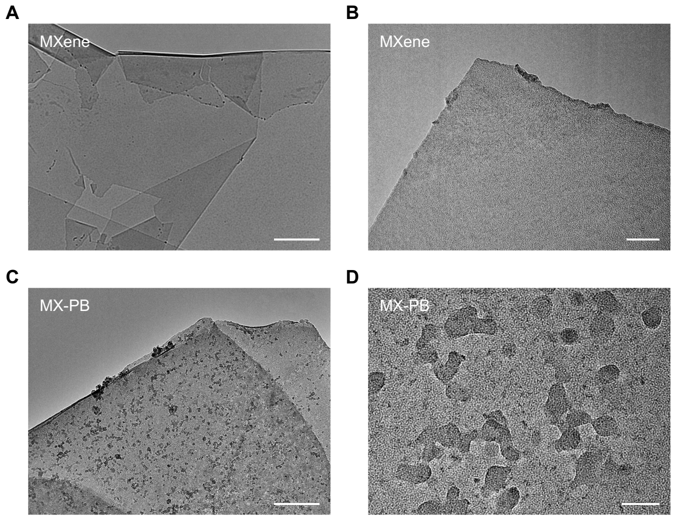

Fig. S18. Characterization of MXene and MX-PB. (**A** to **D**) Bright field transmission electron microscopy (TEM) and magnified TEM images of MXene nanosheet (A and B) and MX-PB nanosheet (C and D). Scale bars, 500 nm (A and C) and 50 nm (B and D).

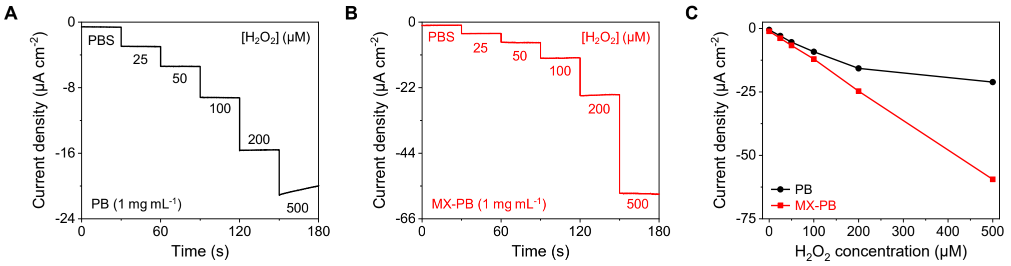

Fig. S19. Characterization of MX-PB as the mediator layer for H_2O_2 detection. (**A** to **C**) Amperometric responses of the H_2O_2 sensors in 0–500 μM H_2O_2 with PB ink (A) and MX-PB (B) as the mediator layer, and the corresponding calibration plots (C). PBS, phosphate-buffered saline.

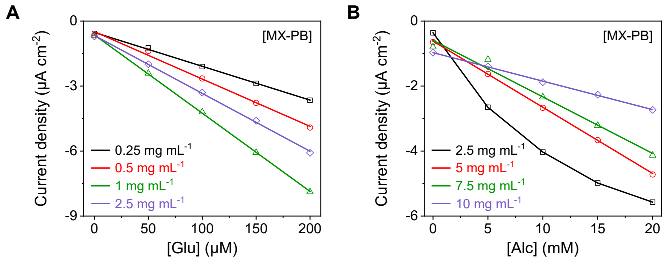

Fig. S20. Optimizations of enzymatic glucose and alcohol sensors. (**A** and **B**) Amperometric calibration plots of the glucose sensor in 0–200 µM Glu (A) and alcohol sensor in 0–20 mM Alc (B) with varying concentrations of MX-PB. Glu, glucose. Alc, alcohol.

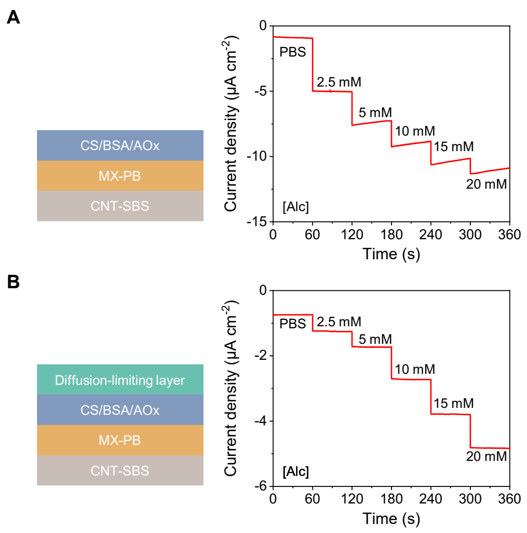

Fig. S21. Evaluation of the diffusion-limiting layer for alcohol sensing. (**A** and **B**) The amperometric responses in 0–20 mM Alc without (A) and with (B) a diffusion-limiting layer. CS, chitosan. BSA, bovine serum albumin.

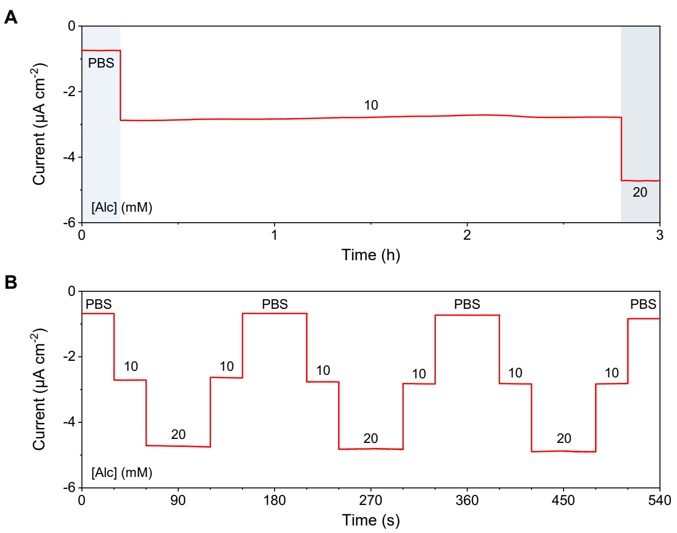

Fig. S22. Long-term stability and repeatability of enzymatic alcohol sensors. (**A**) The long-term stability of alcohol sensor. (**B**) The repeatability of alcohol sensor at varying alcohol concentrations.

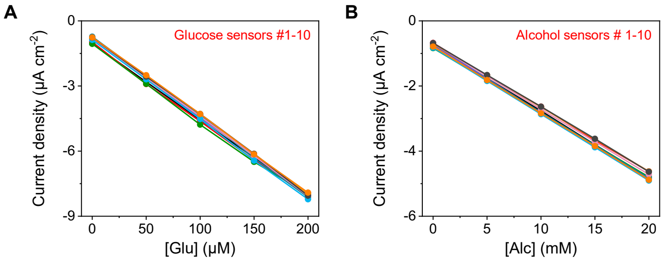

Fig. S23. Reproducibility of the enzymatic sensors. (**A** and **B**) Amperometric calibration plots of 10 individual glucose sensors (A) and alcohol sensors (B).

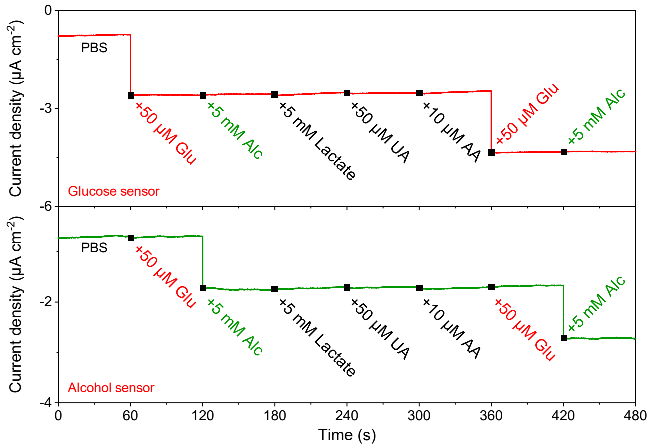

Fig. S24. Selectivity study for enzymatic sensors. Amperometric responses of the glucose sensor and alcohol sensor in response to addition of various common biomarkers found in sweat at physiologically relevant levels. AA, ascorbic acid.

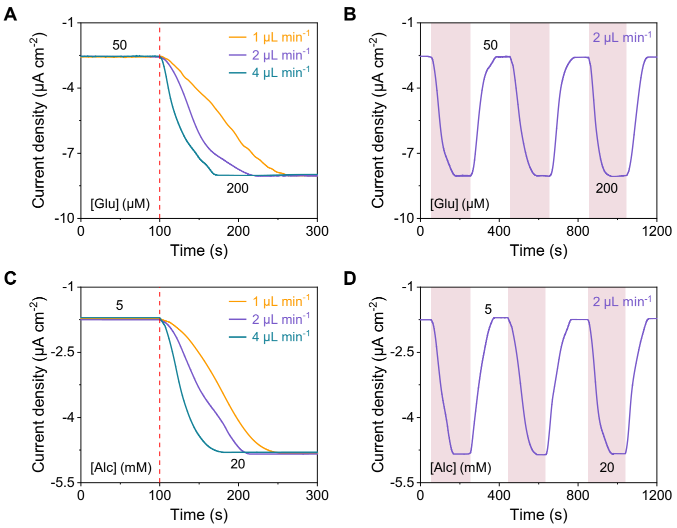

Fig. S25. Evaluation of continuous microfluidic sensing performance under dynamic flow test. (**A** to **D**) Dynamic amperometric responses of glucose sensor (A and B) and alcohol sensor (C and D) under varying flow rates of 1−4 μL min^{-1} (A and C) and after switching inflow solutions with a flow rate of 2 μL min^{-1} (B and D).

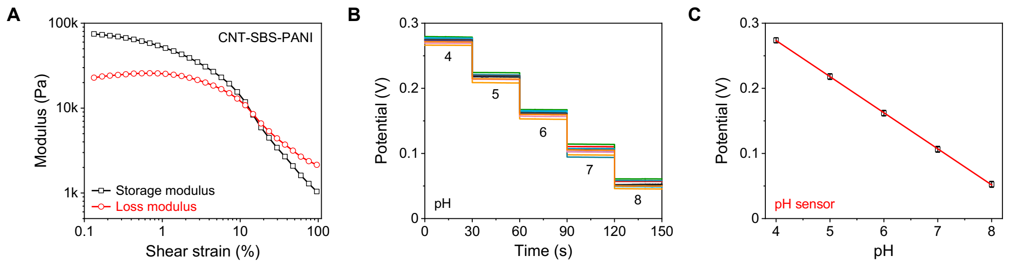

Fig. S26. Characterization of 3D-printed pH sensors. (**A**) Storage and loss modulus as a function of shear strain of CNT-SBS-PANI ink for pH sensor preparation. (**B** and **C**) Batch-to-batch variations of pH sensors (B) and the corresponding calibration plot (C). Error bars represent the s.d. from 10 sensors.

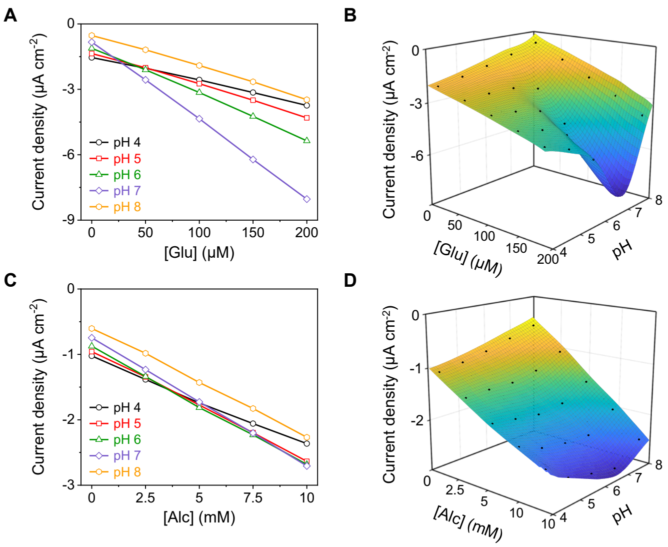

Fig. S27. Influence of pH levels on the responses of glucose and alcohol sensors. (**A** to **D**) Amperometric sensor responses with varying pH levels and the corresponding 3D calibration plots for glucose sensor (A and B) and alcohol sensor (C and D).

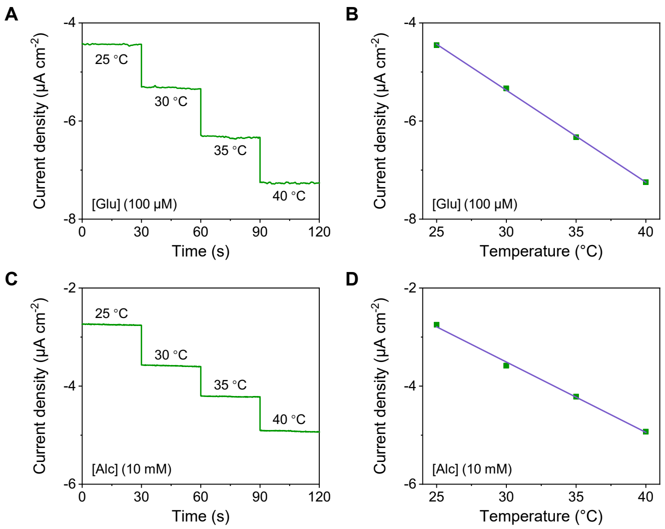

Fig. S28. Influence of temperature on glucose and alcohol sensors. (**A** and **B**) Amperometric sensor response of glucose sensor in 100 μM Glu under varying temperature (A) and the corresponding calibration plot (B). (**C** and **D**) Amperometric sensor response of alcohol sensor in 10 mM Alc under varying temperature (C) and the corresponding calibration plot (D).

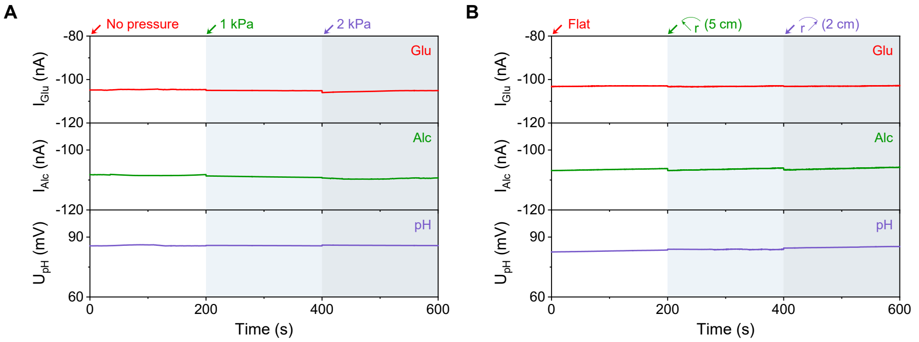

Fig. S29. Mechanical stability of the biochemical sensors. (**A** and **B**) Responses of the biochemical sensors in PBS buffer containing 50 μM Glu and 10 mM Alc under different applied pressure (A) and bending conditions (B).

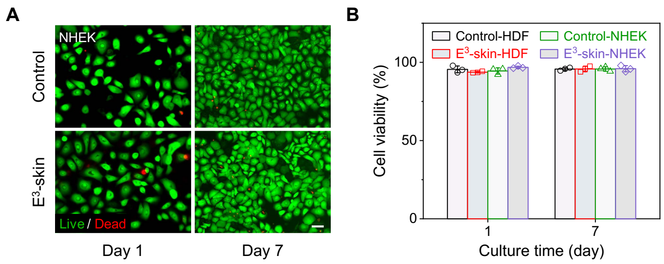

Fig. S30. Biocompatibility evaluation of the 3D-printed e^3-skin. (**A**) Representative live (green)/dead (red) images of normal human epidermal keratinocytes (NHEK) cells seeded on the e^3-skin and in PBS (control) after 1-day and 7-day culture. Scale bar, 50 µm. (**B**) Quantitative analysis of cell viability images over a 7-day period post culture. Error bars represent the s.d. from 3 measurements. HDF, human dermal fibroblasts.

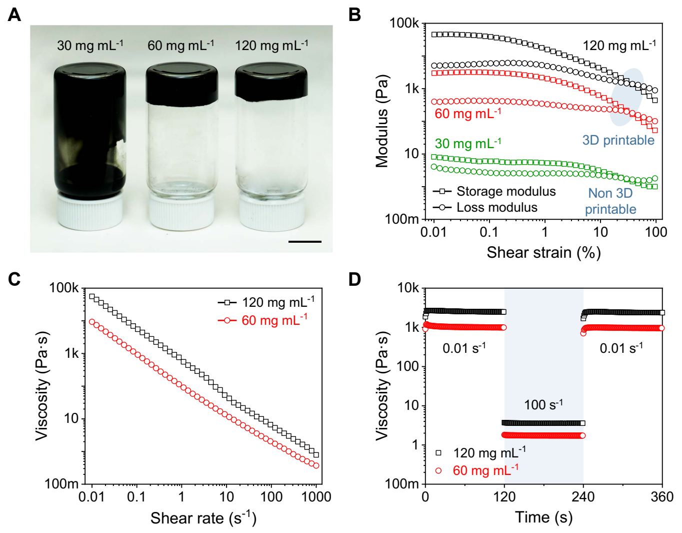

Fig. S31. Characterization of MXene inks. (**A**) Optical image of the MXene inks with concentrations of 30 mg mL^{-1} (L), 60 mg mL^{-1} (M), and 120 mg mL^{-1}(H). Scale bar, 1 cm. (**B**) Storage and loss modulus of MXene inks with different concentrations as a function of shear strain. (**C** and **D**) Viscosity of the MXene inks (M and H) as a function of shear rate (C) and interval shearing time (D). Alternating shear rates between 0.01 s^{-1} and 100 s^{-1} was used to simulate the extrusion process.

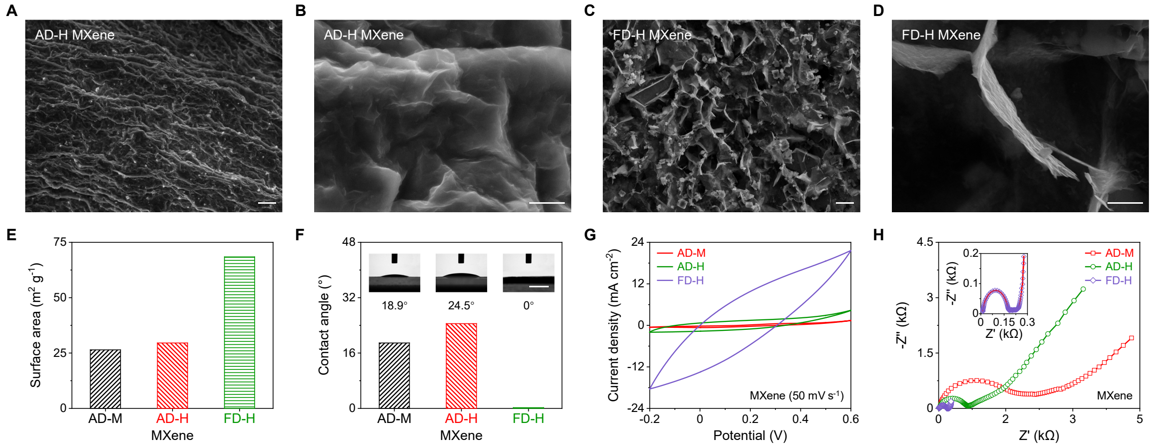

Fig. S32. Characterization of 3D-printed MXene electrodes after different post-treatments. (**A** to **D**) SEM images of highly concentrated MXene after air-drying (AD-H) (A and B) and freeze-drying (FD-H) (C and D) post-treatments. Scale bars, 5 μm (A and C) and 1 μm (B and D). (**E** and **F**) Surface area (E) and contact angle (F) of the different MXene electrodes. AD-M MXene, air-dried MXene with moderate concentration (60 mg mL^{-1}). Scale bar, 1 mm. (**G** and **H**) CV curves at the scan rate of 50 mV s^{-1} (G) and electrochemical impedance spectroscopy (EIS) plots (H) of different MXene electrodes in a solution containing 5 mM $[Fe(CN)_6]^{3-}$ and 0.1 M KCl.

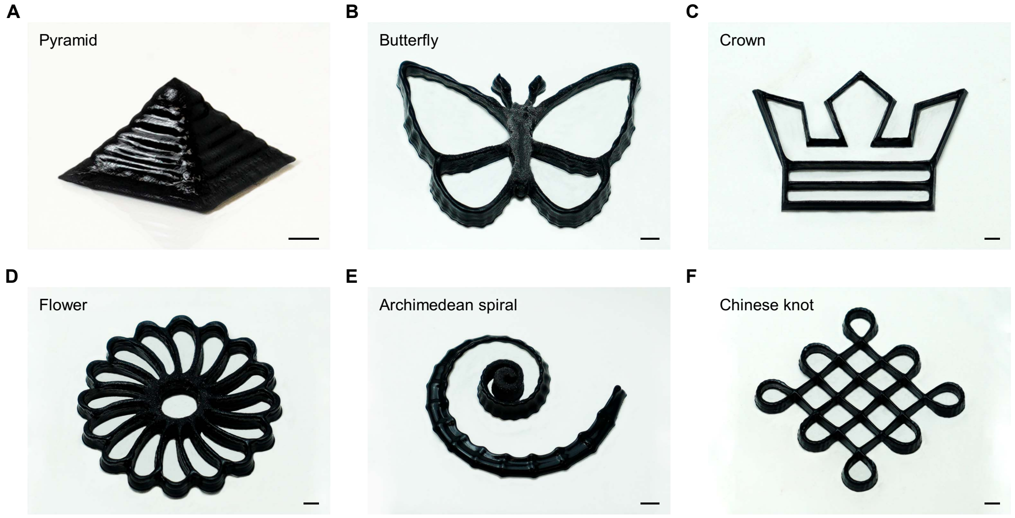

Fig. S33. 3D-printed architectures using high-concentrated MXene inks. (**A** to **F**) Optical images of 3D-printed pyramid (A), butterfly (B), crown (C), flower (D), Archimedean spiral (E), and Chinese knot (F). Scale bars, 1 mm.

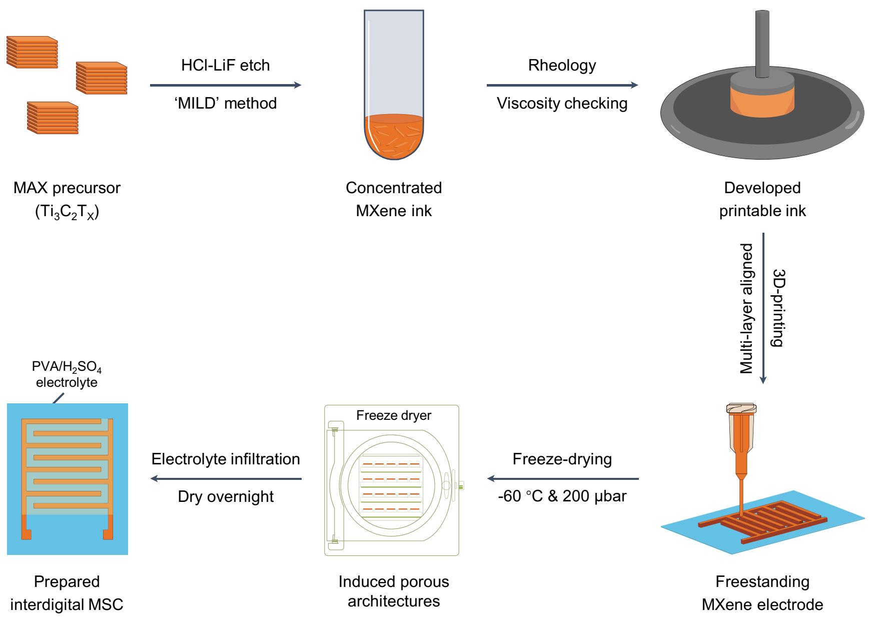

Fig. S34. Schematic illustration of the manufacturing strategy for 3D-printed symmetric interdigital micro-supercapacitor (MSC). Synthesis of 2D MXene nanosheets using the 'MILD' method; development of high-concentrated MXene ink and evaluation of its rheological properties; SSE-based 3D printing of multilayered MXene with an interdigital structure, followed by freeze-drying phase elimination process to induce 3D freestanding porous architectures; preparation of the solid-state symmetrical MSC by printing PVA-H_2SO_4 gel electrolyte.

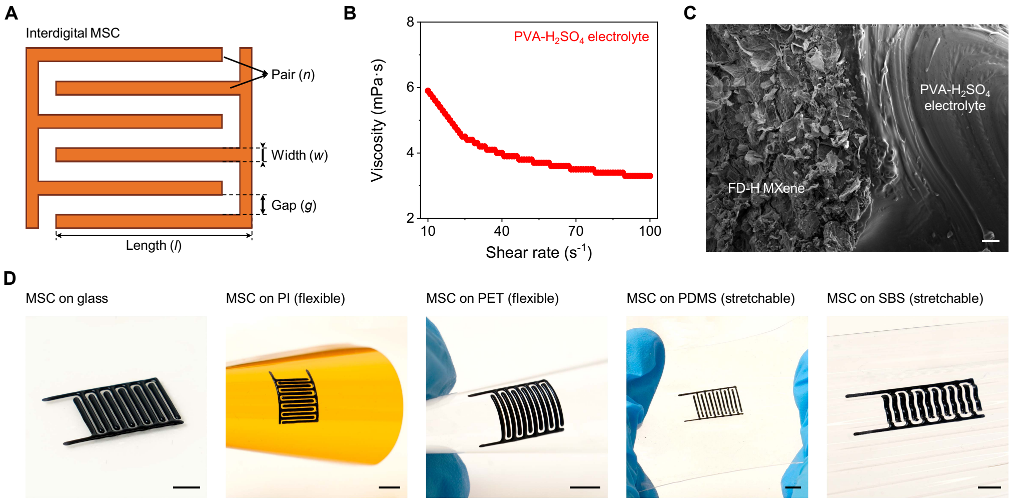

Fig. S35. Characterization of the 3D-printed MSC. (**A**) Schematic illustration of the MSC with interdigital design. (**B**) Viscosity of the PVA-H_2SO_4 gel electrolyte as a function of shear rate. (**C**) Cross-sectional SEM image at the interface between the MXene electrode and PVA-H_2SO_4 gel electrolyte. Scale bar, 10 µm. (**D**) Optical images of 3D-printed MSCs on different substrates. Scale bars, 5 mm. PI, polyimide; PET, polyethylene terephthalate.

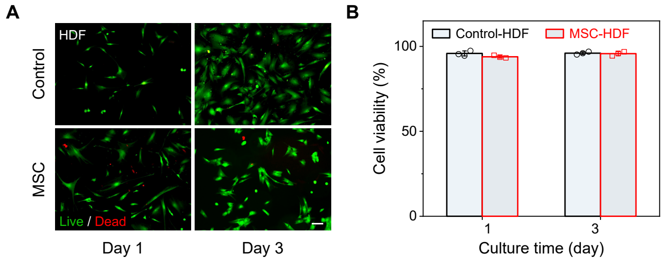

Fig. S36. Biocompatibility evaluation of 3D-printed MSC. (**A**) Representative live (green)/dead (red) images of HDF cells seeded on the MSC and in PBS (control) after 1-day and 3-day culture. Scale bar, 100 μm. (**B**) Quantitative analysis of cell viability images over a 3-day period post culture. Error bars represent the s.d. from 3 measurements.

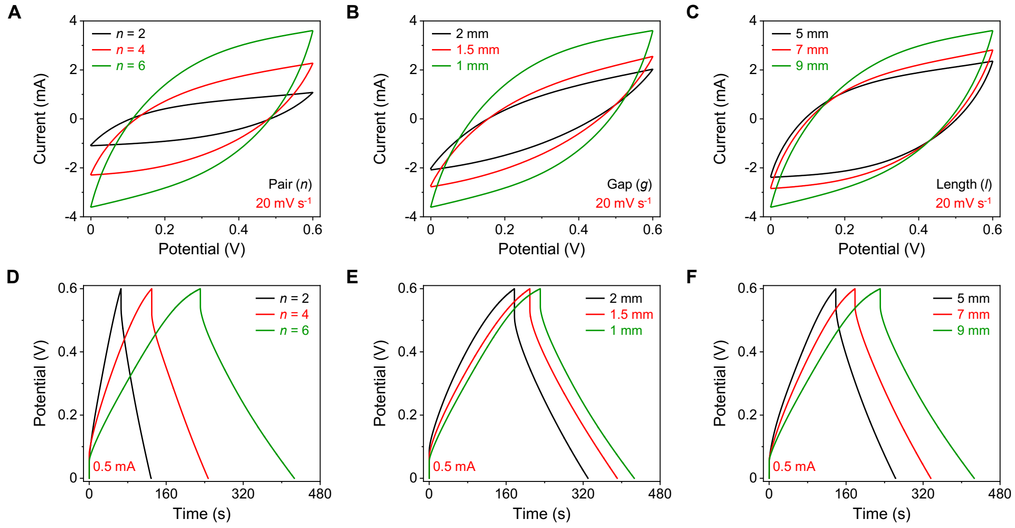

Fig. S37. Parametric study on the electrochemical performance of 3D-printed MSCs. (**A** to **C**) CV curves of 3D-printed MSCs with different number of interdigital pairs (*n*) (A), different gap (*g*) (B), and different length (*l*) (C) at a scan rate of 20 mV s^{-1}. (**D** to **F**) Galvanostatic charge-discharge (GCD) profiles of 3D-printed MSCs with different number of interdigital pairs (*n*) (D), different gap (*g*) (E), and different length (*l*) (F) using a charging-discharging current of 0.5 mA.

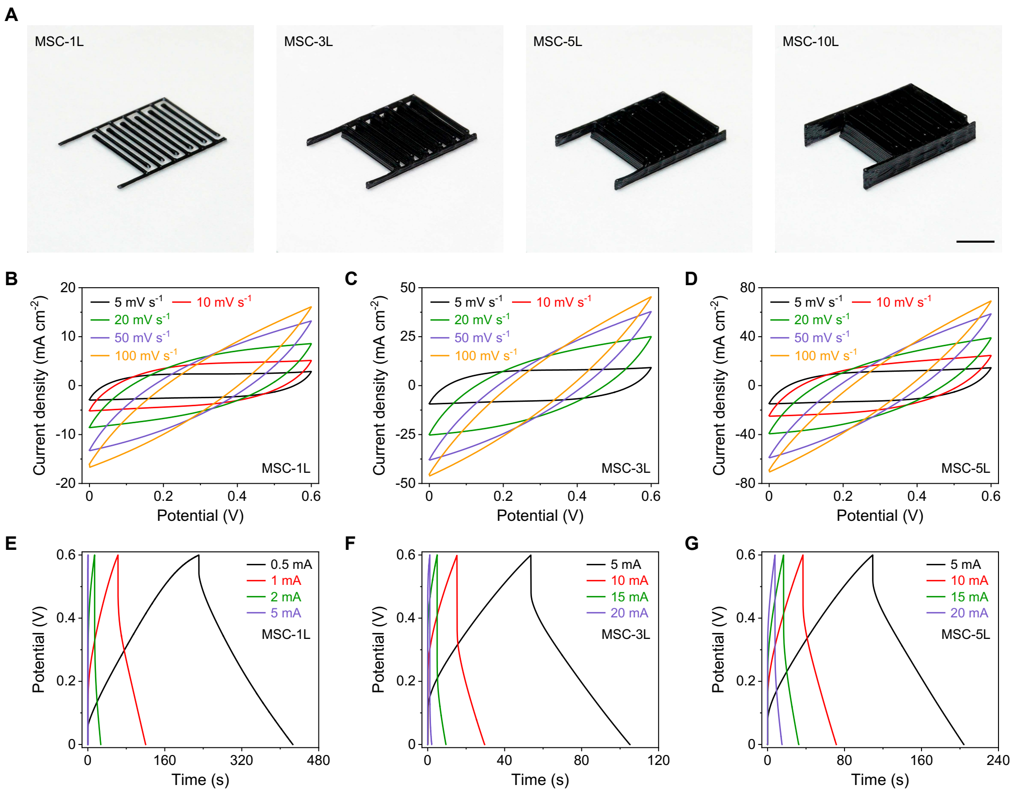

Fig. S38. Electrochemical performance of multilayered 3D-printed MSCs. (**A**) Optical images of MXene-based MSCs with 1 layer (MSC-1L), 3 layers (MSC-3L), 5 layers (MSC-5L), and 10 layers (MSC-10L). Scale bar, 5 mm. (**B** to **D**) CV curves of 3D-printed MSC-1L (B), MSC-3L (C), and MSC-5L (D) at different scan rates. (**E** to **G**) GCD profiles of 3D-printed MSC-1L (E), MSC-3L (F), and MSC-5L (G) using different charging-discharging currents.

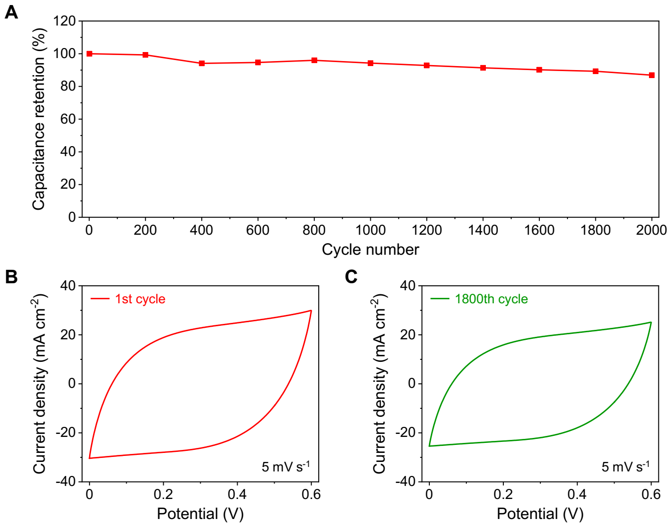

Fig. S39. Long-term cycling stability of the 3D-printed MSC. (**A**) Capacitance retention of the 3D-printed MSC-10L during 2,000 cycles. (**B** and **C**) CV curves of the 3D-printed MSC-10L during the first cycle (B) and the 1800th cycle (C) at the scan rate of 5 mV s^{-1}.

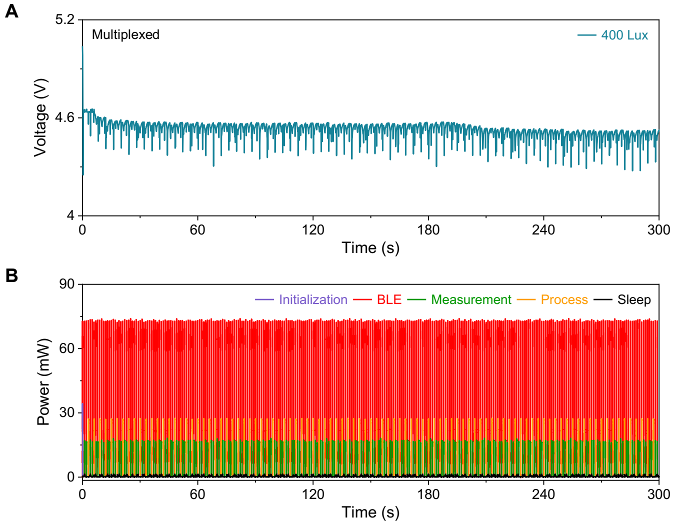

Fig. S40. Characterization of the solar cell-powered e^3-skin performing multiplexed on-body measurement sequences under indoor light intensity. (**A** and **B**) Charging-discharging curve of the MSC (A) and the expanded view of power consumption of multiplexed measurements (B) during a 300s period with a 1 s measurement interval (3 s per data set), and BLE connection interval of 1 s.

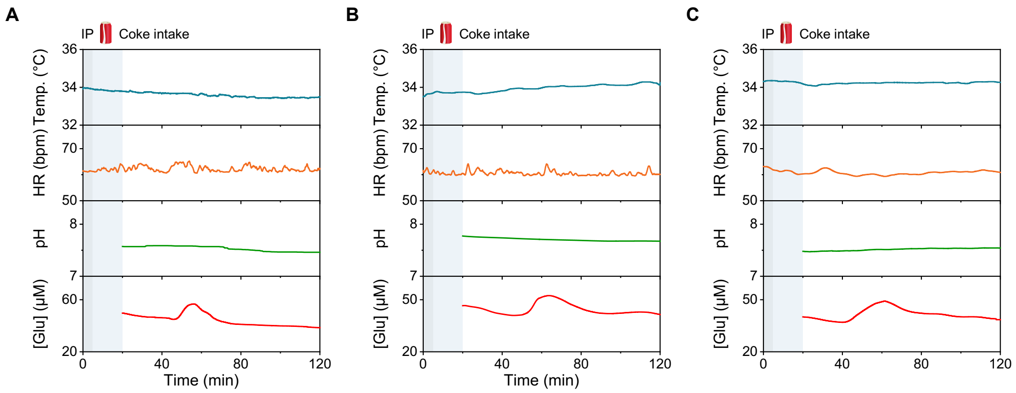

Fig. S41. On-body glucose tolerance study. (**A** to **C**) On-body evaluation of the e^3-skin for multiplexed physiological monitoring during and after soft drink intake on subject 1 (A), subject 2 (B) and subject 3 (C). HR, heart rate; bpm, beats per minute.

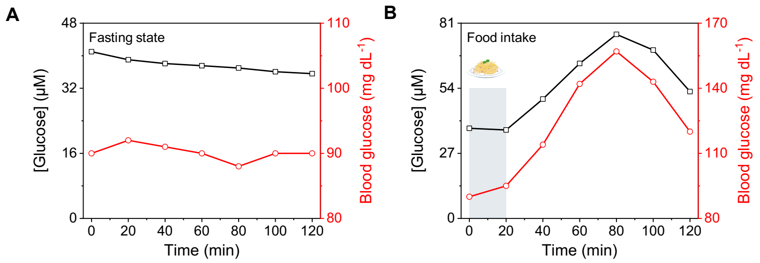

Fig. S42. *In vitro* glucose sensor validation. (**A** and **B**) Dynamic responses of blood glucose levels using a commercial blood-glucose meter and sweat glucose readings obtained from the e^3-skin at fasting state (A) and after food intake (B).

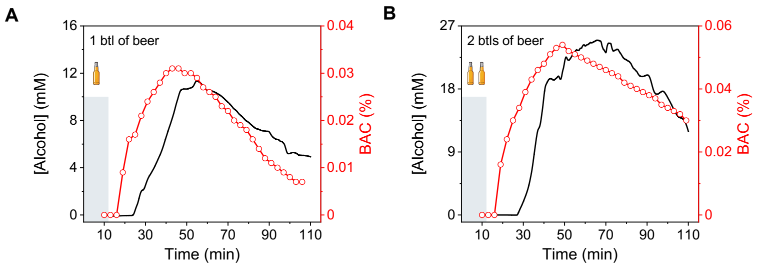

Fig. S43. ***In vivo*** **alcohol sensor validation.** (**A** and **B**) Dynamic responses of blood alcohol content (BAC) using a commercial breathalyser and real-time sweat alcohol readings obtained from the e^3-skin after consumption of 1 bottle (A) and 2 bottles (B) of beer.

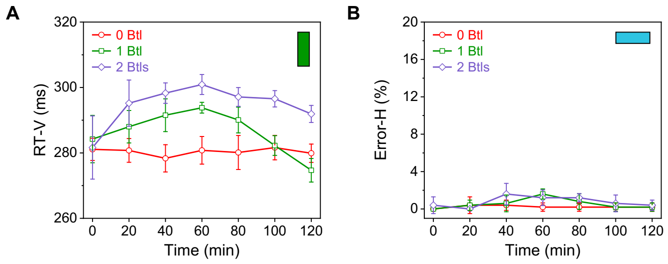

Fig. S44. Influence of alcohol on the reaction time and degree of impairment for control inhibition in cued Go/No-Go task. (**A** and **B**) Mean reaction time to Go targets with vertical cue (RT-V) (A) and commission errors (%) to No-Go targets with horizontal cues (Error-H) (B) for 5 subjects under three alcohol conditions. Error bars represent the s.d. from 5 measurements.

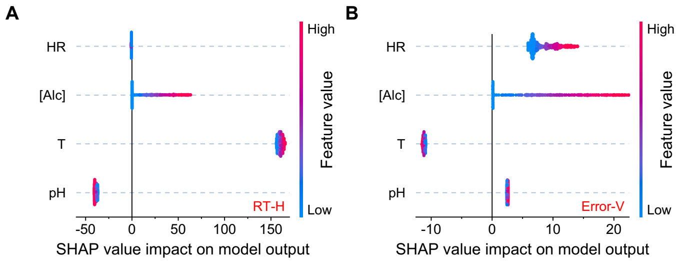

Fig. S45. Shapley additive explanation (SHAP) summary plots for the cued Go/No-Go task based on dataset from the e^3-skin. (**A** and **B**) SHAP summary plot explaining the feature importance in the regression model of RT-H (A) and Error-V (B) for every data point in the testing dataset using all four features.

Table S1. Customizable inks for e^3-skin fabrication

Customizable inks	Printed components	Ink formulation
MXene	Biophysical sensors & interconnects	60 mg mL^{-1} MXene in water
MXene	Micro-supercapacitor electrodes	120 mg mL^{-1} MXene in water
CNT-PDMS	Pressure sensor	3 wt% CNT with PDMS in toluene (1:4)
Ag	Reference electrode	Commercial Ag paint
CNT-SBS	Working & counter electrode	9 wt% CNT + 7 wt% PEG + 25 wt% SBS in toluene
CNT-SBS-PANI	pH sensor	9 wt% CNT + 5 wt% PANI + 7 wt% PEG + 25 wt% SBS in toluene
SBS	Substrate & microfluidics	25 wt% SBS in toluene
MX-PB	Sensing layer	1 & 10 mg mL^{-1} MX-PB in water
Carbagel	Iontophoresis	2 wt% agarose + 5 wt% gelatin + 1 wt% carbachol/KCl in water
PVA-H_2SO_4	Gel electrolyte	10 wt% PVA + 10 wt% H_2SO_4 in water
GOx	Enzymatic layer	1 wt% chitosan in 0.1 M acetic acid + 10 mg mL^{-1} GOx in PBS (2:1)
PU	Diffusion-limiting layer	2 wt% DMF + 3 wt% PU in THF
AOx	Enzymatic layer	1 wt% chitosan in 0.1 M acetic acid + 10 mg mL^{-1} BSA in PBS + AOx (1:1:8)

Video Captions

Video S1. 3D-printed biophysical sensors.

Video S2. 3D-printed microfluidics

Video S3. *In vivo* iontophoresis & microfluidic flow test.

Video S4. 3D-printed micro-supercapacitor.

Video S5. 3D-printed multiple architectures.

REFERENCES AND NOTES

1. T. Ray, J. Choi, A. Bandodkar, S. Krishnan, P. Gutruf, L. Tian, R. Ghaffari, J. Rogers, Bio-integrated wearable systems: A comprehensive review. *Chem. Rev.* **119**, 5461–5533 (2019).

2. Y. Yang, W. Gao, Wearable and flexible electronics for continuous molecular monitoring. *Chem. Soc. Rev.* **48**, 1465–1491 (2019).

3. T. Someya, Z. Bao, G. G. Malliaras, The rise of plastic bioelectronics. *Nature* **540**, 379–385 (2016).

4.C. Xu, Y. Yang, W. Gao, Skin-interfaced sensors in digital medicine: From materials to applications. *Matter* **2**, 1414–1445 (2020).

5. H. Ates, P. Nguyen, L. Gonzalez-Macia, E. Morales-Narváez, F. Güder, J. Collins, C. Dincer, End-to-end design of wearable sensors. *Nat. Rev. Mater.* **7**, 887–907 (2022).

6. Y. Luo, M.R. Abidian, J.H. Ahn, D. Akinwande, A.M. Andrews, M. Antonietti, Z. Bao, M. Berggren, C.A. Berkey, C.J. Bettinger, J. Chen, P. Chen, W. Cheng, X. Cheng, S.J. Choi, A. Chortos, C. Dagdeviren, R.H. Dauskardt, C.A. di, M.D. Dickey, X. Duan, A. Facchetti, Z. Fan, Y. Fang, J. Feng, X. Feng, H. Gao, W. Gao, X. Gong, C.F. Guo, X. Guo, M.C. Hartel, Z. He, J.S. Ho, Y. Hu, Q. Huang, Y. Huang, F. Huo, M.M. Hussain, A. Javey, U. Jeong, C. Jiang, X. Jiang, J. Kang, D. Karnaushenko, A. Khademhosseini, D.H. Kim, I.D. Kim, D. Kireev, L. Kong, C. Lee, N.E. Lee, P.S. Lee, T.W. Lee, F. Li, J. Li, C. Liang, C.T. Lim, Y. Lin, D.J. Lipomi, J. Liu, K. Liu, N. Liu, R. Liu, Y. Liu, Y. Liu, Z. Liu, Z. Liu, X.J. Loh, N. Lu, Z. Lv, S. Magdassi, G.G. Malliaras, N. Matsuhisa, A. Nathan, S. Niu, J. Pan, C. Pang, Q. Pei, H. Peng, D. Qi, H. Ren, J.A. Rogers, A. Rowe, O.G. Schmidt, T. Sekitani, D.G. Seo, G. Shen, X. Sheng, Q. Shi, T. Someya, Y. Song, E. Stavrinidou, M. Su, X. Sun, K. Takei, X.M. Tao, B.C.K. Tee, A.V.Y. Thean, T.Q. Trung, C. Wan, H. Wang, J. Wang, M. Wang, S. Wang, T. Wang, Z.L. Wang, P.S. Weiss, H. Wen, S. Xu, T. Xu, H. Yan, X. Yan, H. Yang, L. Yang, S. Yang, L. Yin, C. Yu, G. Yu, J. Yu, S.H. Yu, X. Yu, E. Zamburg, H. Zhang, X. Zhang, X. Zhang, X. Zhang, Y. Zhang, Y. Zhang, S. Zhao, X. Zhao, Y. Zheng, Y.Q. Zheng, Z. Zheng, T.

Zhou, B. Zhu, M. Zhu, R. Zhu, Y. Zhu, Y. Zhu, G. Zou, X. Chen, Technology roadmap for flexible sensors. *ACS Nano* **17**, 5211–5295 (2023).

7. A. Libanori, G. Chen, X. Zhao, Y. Zhou, J. Chen, Smart textiles for personalized healthcare. *Nat. Electron.* **5**, 142–156 (2022).

8. W. Gao, S. Emaminejad, H. Y. Y. Nyein, S. Challa, K. Chen, A. Peck, H. M. Fahad, H. Ota, H. Shiraki, D. Kiriya, D. H. Lien, G. A. Brooks, R. W. Davis, A. Javey, Fully integrated wearable sensor arrays for multiplexed in situ perspiration analysis. *Nature* **529**, 509–514 (2016).

9. J. Kim, A. Campbell, B. de Ávila, J. Wang, Wearable biosensors for healthcare monitoring. *Nat. Biotechnol.* **37**, 389–406 (2019).

10. J. Heikenfeld, A. Jajack, B. Feldman, S. Granger, S. Gaitonde, G. Begtrup, B. Katchman, Accessing analytes in biofluids for peripheral biochemical monitoring. *Nat. Biotechnol.* **37**, 407–419 (2019).

11. M. Bariya, H. Y. Y. Nyein, A. Javey, Wearable sweat sensors. *Nat. Electron.* **1**, 160–171 (2018).

12. A. J. Bandodkar, W. J. Jeang, R. Ghaffari, J. A. Rogers, Wearable sensors for biochemical sweat analysis. *Annu. Rev. Anal. Chem.* **12**, 1–22 (2019).

13. H. Lee, T. K. Choi, Y. B. Lee, H. R. Cho, R. Ghaffari, L. Wang, H. J. Choi, T. D. Chung, N. Lu, T. Hyeon, S. H. Choi, D. H. Kim, A graphene-based electrochemical device with thermoresponsive microneedles for diabetes monitoring and therapy. *Nat. Nanotechnol.* **11**, 566–572 (2016).

14. Y. Yang, Y. Song, X. Bo, J. Min, O. S. Pak, L. Zhu, M. Wang, J. Tu, A. Kogan, H. Zhang, T. K. Hsiai, Z. Li, W. Gao, A laser-engraved wearable sensor for sensitive detection of uric acid and tyrosine in sweat. *Nat. Biotechnol.* **38**, 217–224 (2020).

15. M. Wang, Y. Yang, J. Min, Y. Song, J. Tu, D. Mukasa, C. Ye, C. Xu, N. Heflin, J. McCune, T. Hsiai, Z. Li, W. Gao, A wearable electrochemical biosensor for the monitoring of metabolites and nutrients. *Nat. Biomed. Eng.* **6**, 1225–1235 (2022).

16. J. Sempionatto, M. Lin, L. Yin, E. De Lapaz, K. Pei, T. Sonsa-ard, A. de Loyola Silva, A. Khorshed, F. Zhang, N. Tostado, S. Xu, J. Wang, An epidermal patch for the simultaneous monitoring of haemodynamic and metabolic biomarkers. *Nat. Biomed. Eng.* **5**, 737–748 (2021).

17. J. Sempionatto, J. Lasalde-Ramírez, K. Mahato, J. Wang, W. Gao, Wearable chemical sensors for biomarker discovery in the omics era. *Nat. Rev. Chem.* **6**, 899–915 (2022).

18. J. Min, J. Tu, C. Xu, H. Lukas, S. Shin, Y. Yang, S. Solomon, D. Mukasa, W. Gao, Skin-interfaced wearable sweat sensors for precision medicine. *Chem. Rev.* **123**, 5049–5138 (2023).

19. R. M. Torrente-Rodriguez, J. Tu, Y. Yang, J. Min, M. Wang, Y. Song, Y. Yu, C. Xu, C. Ye, W. W. IsHak, W. Gao, Investigation of cortisol dynamics in human sweat using a graphene-based wireless mHealth system. *Matter* **2**, 921–937 (2020).

20. J. Tu, J. Min, Y. Song, C. Xu, J. Li, J. Moore, J. Hanson, E. Hu, T. Parimon, T. Wang, E. Davoodi, T. Chou, P. Chen, J. Hsu, H. Rossiter, W. Gao, A wireless patch for the monitoring of C-reactive protein in sweat. *Nature Biomed. Eng.* (2023).

21. S. Mannsfeld, B. Tee, R. Stoltenberg, C. Chen, S. Barman, B. Muir, A. Sokolov, C. Reese, Z. Bao, Highly sensitive flexible pressure sensors with microstructured rubber dielectric layers. *Nat. Mater.* **9**, 859–864 (2010).

22. G. Schwartz, B. Tee, J. Mei, A. Appleton, D. Kim, H. Wang, Z. Bao, Flexible polymer transistors with high pressure sensitivity for application in electronic skin and health monitoring. *Nat. Commun.* **4**, 1859 (2013).

23. R. Truby, J. Lewis. Printing soft matter in three dimensions. *Nature* **540**, 371–378 (2016).

24. M. Saadi, A. Maguire, N. Pottackal, S. Thakur, M. Ikram, A. Hart, P. Ajayan, M. Rahman, Direct ink writing: A 3D printing technology for diverse materials. *Adv. Mater.* **34**, 2108855 (2022).

25. X. Mu, T. Bertron, C. Dunn, H. Qiao, J. Wu, Z. Zhao, C. Saldana, H. J. Qi, Porous polymeric materials by 3D printing of photocurable resin. *Mater. Horiz.* **4**, 442–449 (2017).

26. N. Kleger, M. Cihova, K. Masania, A. Studart, J. Löffler, 3D printing of salt as a template for magnesium with structured porosity. *Adv. Mater.* **31**, 1903783 (2019).

27. X. Zan, X. Wang, K. Shi, Y. Feng, J. Shu, J. Liao, R. Wang, C. Peng, S. Magdassi, X. Wang, Three-dimensional porous tungsten via DLP 3D printing from transparent ink. *J. Phys. D Appl. Phys.* **55**, 444004 (2022).

28. C. Nyitray, R. Chang, G. Faleo, K. Lance, D. Bernards, Q. Tang, T. Desai, Polycaprolactone thin-film micro- and nanoporous cell-encapsulation devices. *ACS Nano* **9**, 5675–5682 (2015).

29. M. Naguib, M. Kurtoglu, V. Presser, J. Lu, J. Niu, M. Heon, L. Hultman, Y. Gogotsi, M. Barsoum, Two-dimensional nanocrystals produced by exfoliation of Ti_3AlC_2. *Adv. Mater.* **23**, 4248–4253 (2011).

30. M. Ghidiu, M. Lukatskaya, M. Zhao, Y. Gogotsi, M. Barsoum, Conductive two-dimensional titanium carbide ‘clay’ with high volumetric capacitance. *Nature* **516**, 78–81 (2014).

31. M. Naguib, V. Mochalin, M. Barsoum, Y. Gogotsi, 25th Anniversary Article: MXenes: A new family of two-dimensional materials. *Adv. Mater.* **26**, 992–1005 (2014).

32. A. Karyakin, E. Karyakina, L. Gorton, On the mechanism of H_2O_2 reduction at Prussian blue modified electrodes. *Electrochem. Commun.* **1**, 78–82 (1999).

33. V. Kondratiev, A. Tikhomirova, V. Malev, Study of charge transport processes in Prussian-blue film modified electrodes. *Electrochim. Acta* **45**, 751–759 (1999).

34. Y. Song, J. Min, Y. Yu, H. Wang, Y. Yang, H. Zhang, W. Gao, Wireless battery-free wearable sweat sensor powered by human motion. *Sci. Adv.* **6**, eaay9842 (2020).

35. Y. Yu, J. Nassar, C. Xu, J. Min, Y. Yang, A. Dai, R. Doshi, A. Huang, Y. Song, R. Gehlhar, A. Ames, W. Gao, Biofuel-powered soft electronic skin with multiplexed and wireless sensing for human-machine interfaces. *Sci. Robot.* **5**, eaaz7946 (2020).

36. Y. Song, D. Mukasa, H. Zhang, W. Gao, Self-powered wearable biosensors. *Acc. Mater. Res.* **2**, 184–197 (2021).

37. J. Min, S. Demchyshyn, J. Sempionatto, Y. Song, B. Hailegnaw, C. Xu, Y. Yang, S. Solomon, C. Putz, L. Lehner, J. Schwarz, C. Schwarzinger, M. Scharber, M. Kaltenbrunner, W. Gao, An autonomous wearable biosensor powered by a perovskite solar cell. *Nat. Electron.* (2023).

38. X. Ding, Y. Zhao, C. Hu, Y. Hu, Z. Dong, N. Chen, Z. Zhang, L. Qu, Spinning fabrication of graphene/polypyrrole composite fibers for all-solid-state, flexible fibriform supercapacitors. *J. Mater. Chem. A* **2**, 12355–12360 (2014).

39. Y. Shao, L. Wei, X. Wu, C. Jiang, Y. Yao, B. Peng, H. Chen, J. Huangfu, Y. Ying, C. Zhang, J. Ping, Room-temperature high-precision printing of flexible wireless electronics based on MXene inks. *Nat. Commun.* **13**, 3223 (2022).

40. J. Orangi, F. Hamade, V. Davis, M. Beidaghi, 3D printing of additive-free 2D $Ti_3C_2T_x$ (MXene) ink for fabrication of micro-supercapacitors with ultra-high energy densities. *ACS Nano* **14**, 640–650 (2020).

41. K. Li, J. Zhao, A. Zhussupbekova, C. Shuck, L. Hughes, Y. Dong, S. Barwich, S. Vaesen, I. Shvets, M. Möbius, W. Schmitt, Y. Gogotsi, V. Nicolosi, 4D printing of MXene hydrogels for high-efficiency pseudocapacitive energy storage. *Nat. Commun.* **13**, 6884 (2022).

42. X. Li, H. Li, X. Fan, X. Shi, J. Liang, 3D-printed stretchable micro-supercapacitor with remarkable areal performance. *Adv. Energy Mater.* **10**, 1903794 (2020).

43. W. Yang, J. Yang, J. Byun, F. Moissinac, J. Xu, S. Haigh, M. Domingos, M. Bissett, R. Dryfe, S. Barg, 3D printing of freestanding MXene architectures for current-collector-free supercapacitors. *Adv. Mater.* **31**, 1902725 (2019).

44. S. Abdolhosseinzadeh, R. Schneider, A. Verma, J. Heier, F. Nüesch, C. Zhang, Turning trash into treasure: Additive free MXene sediment inks for screen-printed micro-supercapacitors. *Adv. Mater.* **32**, 2000716 (2020).

45. A. Lipatov, H. Lu, M. Alhabeb, B. Anasori, A. Gruverman,Y. Gogotsi, A. Sinitskii, Elastic properties of 2D Ti3C2TxMXene monolayers and bilayers. *Sci. Adv.* **4**, eaat0491 (2018).

46. C. Zhang, L. McKeon, M. Kremer, S. Park, O. Ronan, A. Seral-Ascaso, S. Barwich, C. Coileáin, N. McEvoy, H. Nerl, B. Anasori, J. Coleman, Y. Gogotsi, V. Nicolosi, Additive-free MXene inks and direct printing of micro-supercapacitors. *Nat. Commun.* **10**, 1795 (2019).

47. National Institute on Alcohol Abuse and Alcoholism, Alcohol's effects on health; https://niaaa.nih.gov/alcohols-effects-health.

48. C. Spies, M. Sander, K. Stangl, J. Fernandez-Sola, V. Preedy, E. Rubin, S. Andreasson, E. Hanna, W. Kox. Effects of alcohol on the heart. *Curr. Opin. Crit. Care* **7**, 337–343 (2001).

49. M. Oscar-Berman, K. Marinković, Alcohol: Effects on neurobehavioral functions and the brain. *Neuropsychol. Rev.* **17**, 239–257 (2007).

50. J. O'Keefe, S. Bhatti, A. Bajwa, J. DiNicolantonio, C. Lavie, Alcohol and cardiovascular health: The dose makes the poisonor the remedy. *Mayo Clin. Proc.* **89**, 382–393 (2014).

51.B. Abroms, M. Fillmore, C. Marczinski, Alcohol-induced impairment of behavioral control: Effects on the alteration and suppression of prepotent responses. *J. Stud. Alcohol* **64**, 687–695 (2003).

52. C. Marczinski, B. Abroms, M. Van Selst, M. Fillmore, Alcohol-induced impairment of behavioral control: Differential effects on engaging vs. disengaging responses. *Psychopharmacology (Berl.)* **182**, 452–459 (2005).

53. Y. Yu, J. Li, S. Solomon, J. Min, J. Tu, W. Guo, C. Xu, Y. Song, W. Gao, All-printed soft human-machine interface for robotic physicochemical sensing. *Sci. Robot.* **7**, eabn0495 (2022).

54. Y. Deng, T. Shang, Z. Wu, Y. Tao, C. Luo, J. Liang, D. Han, R. Lyu, C. Qi, W. Lv, F. Kang, Q. Yang, Fast gelation of Ti3C2TxMXene initiated by metal ions. *Adv. Mater.* **31**, 1902432 (2019).

55. E. Satheeshkumar, T. Makaryan, A. Melikyan, H. Minassian, Y. Gogotsi, M. Yoshimura, One-step solution processing of Ag, Au and Pd@MXene hybrids for SERS. *Sci. Rep.* **6**, 32049 (2016).

56. L. Lorencova, T. Bertok, J. Filip, M. Jerigova, D. Velic, P. Kasak, K. Mahmoud, J. Tkac, Highly stable $Ti_3C_2T_x$ (MXene)/Pt nanoparticles-modified glassy carbon electrode for H_2O_2 and small molecules sensing applications. *Sens. Actuators B Chem.* **263**, 360–368 (2018).

57. Y. Jiang, X. Zhang, L. Pei, S. Yue, L. Ma, L. Zhou, Z. Huang, Y. He, J. Gao, Silver nanoparticles modified two-dimensional transition metal carbides as nanocarriers to fabricate acetycholinesterase-based electrochemical biosensor. *Chem. Eng. J.* **339**, 547–556 (2018).

58. Y. He, X. Zhou, L. Zhou, X. Zhang, L. Ma, Y. Jiang, J. Gao, Self-reducing Prussian blue on Ti3C2TxMXene nanosheets as a dual-functional nanohybrid for hydrogen peroxide and pesticide sensing. *Ind. Eng. Chem. Res.* **59**, 15556–15564 (2020).

59. H. Zhang, Z. Wang, F. Wang, Y. Zhang, H. Wang, Y. Liu, Ti_3C_2 MXene mediated Prussian blue in situ hybridization and electrochemical signal amplification for the detection of exosomes. *Talanta* **224**, 121879 (2021).

60. M. Alhabeb, K. Maleski, B. Anasori, P. Lelyukh, L. Clark, S. Sin, Y. Gogotsi, Guidelines for synthesis and processing of two-dimensional titanium carbide (Ti3C2TxMXene). *Chem. Mater.* **29**, 7633–7644 (2017).
